# 16S rRNA gene-based microbiome analysis identifies candidate bacterial strains that increase the storage time of potato tubers

**DOI:** 10.1038/s41598-021-82181-9

**Published:** 2021-02-04

**Authors:** Franziska Buchholz, Robert Junker, Abdul Samad, Livio Antonielli, Nataša Sarić, Tanja Kostić, Angela Sessitsch, Birgit Mitter

**Affiliations:** 1 Center for Health & Bioresources, Bioresources Unit, AIT Austrian Institute of Technology GmbH, Konrad-Lorenz-Strasse 24, 3430 Tulln, Austria; 2 Evolutionary Ecology of Plants, Department of Biology, Philipps-University Marburg, 35043 Marburg, Germany; 3 Department of Biosciences, University of Salzburg, 5020 Salzburg, Austria

**Keywords:** Microbial ecology, Microbiology, Microbial communities

## Abstract

In the past, the potato plant microbiota and rhizosphere have been studied in detail to improve plant growth and fitness. However, less is known about the postharvest potato tuber microbiome and its role in storage stability. The storage stability of potatoes depends on genotype and storage conditions, but the soil in which tubers were grown could also play a role. To understand the ecology and functional role of the postharvest potato microbiota, we planted four potato varieties in five soil types and monitored them until the tubers started sprouting. During storage, the bacterial community of tubers was analysed by next-generation sequencing of the 16S rRNA gene amplicons. The potato tubers exhibited soil-dependent differences in sprouting behaviour. The statistical analysis revealed a strong shift of the tuber-associated bacterial community from harvest to dormancy break. By combining indicator species analysis and a correlation matrix, we predicted associations between members of the bacterial community and tuber sprouting behaviour. Based on this, we identified *Flavobacterium* sp. isolates, which were able to influence sprouting behaviour by inhibiting potato bud outgrowth.

## Introduction

Plant microbiota are known for their importance in the healthy growth and development of the plant^1^. Much research has been carried out to understand the microbiomes of crop plants during vegetative growth, with the goal of improving agricultural productivity^2–5^. In contrast, few studies have been published on the postharvest microbiomes of food crops and their role in storage stability^6–9^, although strategies to reduce food loss are as important in ensuring food supply as an increase in food production.

In 2015, the FAO published an article stating that 45% of roots and tubers produced globally were lost or wasted after harvesting^10^. Post-harvest crop loss and waste is not a new problem but has changed little over the last 40 years^11^. Especially critical is storage, not only in developing countries, where there is often a lack of adequate storage facilities, but also in the industrialized world. Here, agriculture often depends on a single cropping season, and therefore, the year-round availability of fresh produce is dependent upon storage on an industrial scale. Using the example of potato tubers, the UK reported an overall loss of 17% of the annual production of 77,000 tons of potato tubers in 2012, which was caused by premature sprouting and rotting during postharvest storage^12^. The premature sprouting of tubers is one of the major causes of the large quantities of potato loss during storage worldwide. The number of marketable tubers decreases due to factors such as decreased fresh weight due to water loss from sprouted surfaces and the remobilization of starch^13,14^.

Following harvest, potato tubers do not sprout for a certain period of time, even if the environmental conditions are optimal, because they are in a physiological state of deep dormancy^15^. Dormancy means a temporary growth arrest of meristematic tissue, which serves to protect the tubers as the organ of vegetative reproduction of plants during the winter months^15^. The period of potato tuber dormancy is cultivar specific and varies from 2 to 3 months in the case of field-grown potatoes^16^. After the natural period of dormancy comes to an end, the tubers start to sprout.

For a considerable length of time, isopropyl-*N*-(3-chlorophenyl carbamate (chlorpropham or CIPC) has been used to prevent premature sprouting and to ensure potato tuber marketability even after many months of storage. CIPC is still the most effective sprout-suppressing chemical worldwide. Due to its ability to inhibit meristematic cell division, sprout development is delayed in potato tubers during storage^17^. New regulations laid down in 2017 described CIPC and its metabolite as toxic and cancerogenic. Consequently, there has been growing interest in alternative strategies to prevent the premature sprouting of potato tubers during storage.

In addition to the plant genotype and storage conditions, the sprouting behaviour of potato tubers also depends on the field site in which tubers are grown, i.e., tubers of the same potato cultivar grown in different fields often display differences in storage stability (personal communication with Austrian potato producers). Recently, we demonstrated that the soil represents the main reservoir for bacteria-colonizing potato tubers and that the bacteria are recruited largely independently from the potato variety^18^. This implies that the differences in storage stability observed for tubers of the same potato variety that were grown in different soils could at least partly be due to differences in the tuber microbiota.

Furthermore, Slininger et al. reported on the ability of bacteria to inhibit potato tuber sprouting during storage^19^. In this study, tubers of the Russet Burbank variety were sprayed with suspensions of bacteria after having been harvested in two different storage sites and over two consecutive years. During the test period, tubers treated with two strains belonging to *Pseudomonas fluorescens* and *Enterobacter cloacae* had significantly fewer sprouts than the control tubers^19^.

These findings prompted us to test the hypotheses that the bacterial community that colonizes potato tubers influences the storage stability of potatoes; we were also able to identify bacterial taxa that positively or negatively affect tuber sprouting from microbiome data. To do so, we specifically addressed the composition, community dynamics and storage stability of the tuber-associated microbiota. We analysed the bacterial communities of four potato cultivars that were grown in commercial potting soil and four different farmland soils. At maturity, tubers were harvested and used for 16S rRNA gene amplicon sequencing. Subsequently, tubers were stored until sprouting. At different time points (throughout dormancy, at dormancy break and sprouting), tuber samples were used for 16S rRNA gene-based bacterial community analysis to determine the effect of soil, plant genotype and physiological stage on the bacterial community assembly in the potato tuber. By using a correlation analysis, bacterial taxa associated with long storage times were identified, and their effects on potato tuber sprouting were confirmed in further experiments.

## Results

### Sprouting behaviour of potato tubers

In this study, the stored tubers exhibited clear variety-specific differences in sprouting behaviour, with the Agata variety being the earliest and the Hermes variety the latest to sprout. However, within each variety, there were differences in sprouting time depending on the soil in which the tubers had grown (Suppl. Table 1). These soil-dependent differences in sprouting were greatest in the Fabiola variety (sprouting time ranged from 135 to 169 days after harvest) and least significant in the Hermes variety (158 to 168 days after harvest). By examining the storage stability of tubers broken down by soil type, we found that tubers grown in soil from the field site in Karnabrunn sprouted on average up to 9 days later than the tubers from other soils. The chemical profile of the soil collected in Karnabrunn was similar to that of the other farmland soils, especially those from Kettlasbrunn. The soil collected in Tulln showed reduced K and P contents compared to those of the other farmland soils. As expected, the potting soil differed most significantly from the other soils, i.e., there was no clay, and the exchange capacity was substantially higher than that of the farmland soils (Suppl. Table S2). Statistical analysis revealed that the chemical parameters of the soils did not correlate with the sprouting behaviour of tubers (data not shown).

### Sequencing results

The sequencing of 16S rRNA gene amplicons of tubers, sprouts and soil samples yielded 9,158,550 high-quality merged reads, corresponding to an average of 33,920 reads per sample. All sequences were cut to a read length of 360 bp. To ensure sufficient diversity by maintaining an adequate sequencing depth, samples with a low read number were excluded from the analysis. Therefore, the data of four samples were excluded from further analysis (LC_T3_PS; LC_T7_K; H_T6_K: LC_T7_T; H_T6_K; F_T7_K). A detailed explanation for the sample naming can be found in Table 1. Sequencing reads from all 270 samples were clustered into operational taxonomic units (OTUs), which resulted in 11,485 OTUs with an average of 42,537 OTUs per sample.

**Table 1:** Abbreviation of sample names.

	Abbreviation
**Cultivar**	
Agata	A
Fabiola	F
Hermes	H
Lady Claire	LC
**Timepoint**	
Seed potato tuber	T0
First generation	T1
Second generation	T2
Dormancy: 2 weeks after harvesting	T3
Dormancy: 5 weeks after harvesting	T4
Dormancy: 10 weeks after harvesting	T5
Dormancy break	T6
Sprouting	T7
Sprouts	T7_Sprouts
**Soil type**	
Potting soil	PS
Kettlasbrunn A	KA
Kettlasbrunn B	KB
Karnabrunn	K
Tulln	T

### The bacterial community of potato tubers during storage

**Figure 1:**
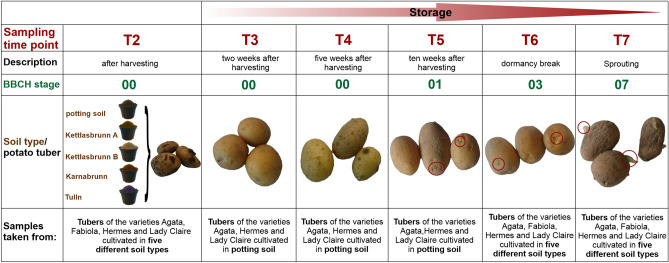
Overview of potato cultivars, sampling time points and corresponding BBCH stages investigated in this study. After harvesting four tuber varieties (Agata, Fabiola, Lady Claire and Hermes) from five different soil types (T2), tubers were stored at 8–10 °C in darkness. After 2 (T3), 5 (T4) and 10 weeks (T5), tubers were sampled. To consider the individual dormancy break (T6) of each variety, tubers were sampled according to the BBCH scale at stage 03. The same procedure was performed for samples that were taken at sprouting at stage 05 (T7). Additionally, at T7, sprout samples were taken. At each sampling time point, tuber/sprout samples were used for 16S rRNA gene amplicon sequencing. The red circles mark sites on the tubers that show visible signs of sprouting.

To test whether the bacterial community in potato tubers changes during storage, the 16S rRNA gene amplicon data of the potato tubers of the Agata, Lady Claire and Hermes varieties, cultivated in five different soil types (T2), stored until dormancy break (T6) and until sprouting (T7) were analysed as well as samples of the sprouts (T7_Sprouts) (Fig. 1). Cultivation of the cultivar Fabiola in the soil Kettlasbrunn B did not yield sufficient tubers for analysis throughout the whole storage period, i.e., no data were available for T7 and the sprouts. Therefore, community data for the Fabiola variety were not included in the following statistical analysis.

For the statistical analysis, we selected only OTUs that showed at least 0.01% relative abundance (1425 OTUs) and that were present in at least two of three replicates (1395 rOTUs). We considered them “reproducibly occurring OTUs” (rOTUs)^2^.

For calculation of alpha diversity values, read numbers were rarefied to 6785 reads in each sample. The permutation ANOVA of rOTU richness (9999 permutations) revealed significant differences in the bacterial community of tubers and sprouts between cultivars, time points and soil types (observed for cultivar (F value = 3.963, P value = 0.0211*), for time point (F value = 4.762, P value = 0.0021*) and for soil type (F value = 3.779, P value = 0.0057**). The same results were obtained with Simpson’s index for the factors cultivar (F value = 4.594, P value = 0.01*), time point (F value = 6.741, P value = 0.0004***) and soil type (F value = 5.123, P value = 0.0008***) (Suppl. Table S3). Overall, the statistical analysis showed that the soil type had the strongest impact on the microbial community in potato tubers, followed by time point and cultivar.

**Figure 2:**
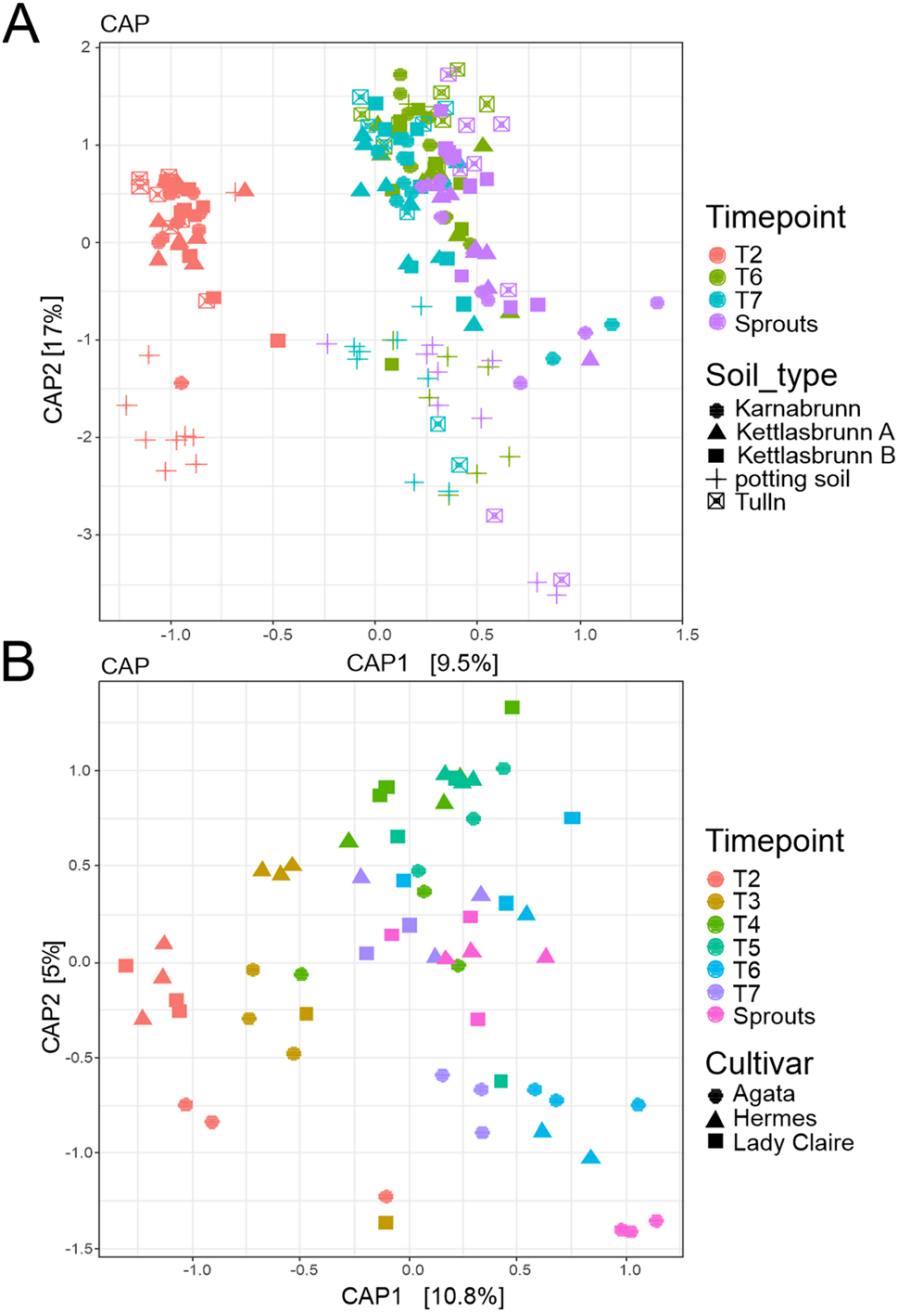
Constrained analysis of principal coordinates (CAP) of Bray–Curtis dissimilarities. CAP based on the V5–V7 regions of the 16S rRNA gene investigated for (**A**) tubers of the varieties (Agata, Lady Claire and Hermes) sampled at T2, T6 and T7 and (**B**) tubers of the varieties (Agata, Lady Claire and Hermes) sampled at T2-7 as well as sprout samples at T7. An overview of potato cultivars, soil types and sampling time points is shown in Fig. 1.

Similarly, the bacterial community composition (beta diversity) in potato tubers was significantly affected by the storage time, soil type and plant variety. The CAP ordination plot indicated a shift in the bacterial community of tubers from harvest to dormancy break and sprouting, which could be proven by PERMANOVA on the Bray–Curtis dissimilarity distance matrix (R2 = 0.113, P value = 0.0004***) (Fig. 2A). Additionally, the bacterial communities in tubers grown in different soil types (R2 = 0.255, P value = 0.0004***) and of different varieties were significantly different from one other (R2 = 0.027, P value = 0.0021**) (Suppl. Table S4). The results for the factor time point (P value = 0.01**), genotype (P value = 0.01**) and soil type (P value = 0.01**) could be confirmed by the multivariate generalized linear model for multivariate abundance data on the Bray–Curtis dissimilarity distance matrix (999 permutations) (Suppl. Table S4).

### The dynamics of bacterial communities in potato tubers during storage

To obtain more in-depth insight into the changes in community composition during storage, we compared the bacterial communities in tubers of the Agata, Lady Claire and Hermes varieties grown in potting soil at six different timepoints: T2 (harvest), T3 (2 weeks after harvesting), T4 (5 weeks after harvesting), T5 (10 weeks after harvesting), T6 (dormancy break), T7 (sprouting) and in the corresponding sprouts (T7_Sprouts) (Fig. 1). We focused here on tubers grown in potting soil, since the cultivation of potatoes in farmland soil did not yield sufficient tubers to be analysed throughout the entire storage period.

**Figure 3:**
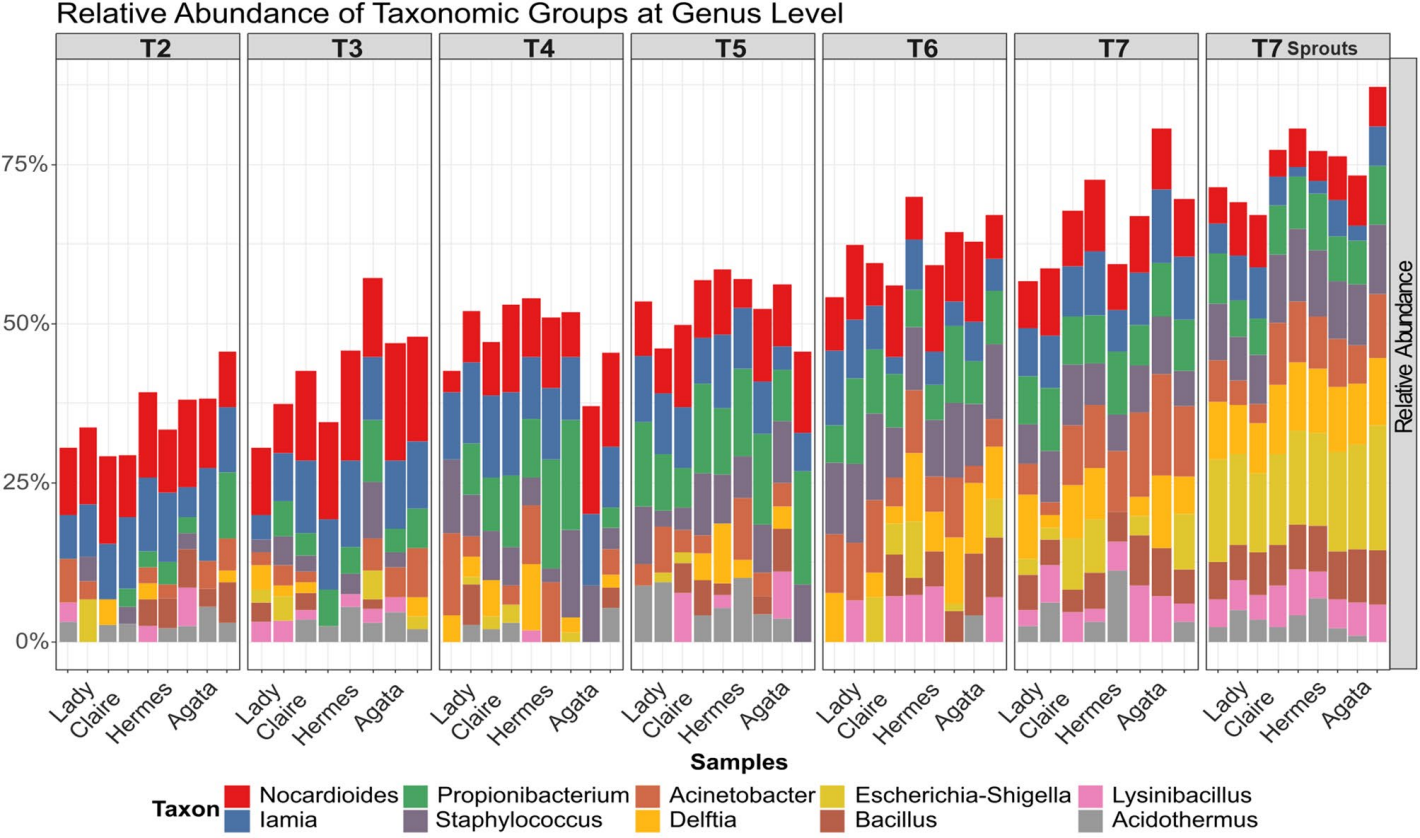
Differentially abundant taxa at the genus level. Visualization of differentially abundant taxonomic groups at the genus level of the bacterial community in tubers of three different potato cultivars (Agata, Hermes and Lady Claire) at all sampling time points T2-7 as well as in sprout samples at T7. Differentially abundant taxa were calculated with the function varSelRF^48^ and visualized in barplot with the function group.abundant.taxa of the R package RAM^47^.

Again, we filtered data for OTUs with at least 0.01% relative abundance and “reproducibly occurring OTUs”. After both filtering steps, 1108 rOTUs remained. Before calculating alpha values, read numbers were rarefied to 6761 reads in each sample. The permutation ANOVA of richness and evenness (9999 permutations) did not reveal differences in the alpha diversity of the tuber microbiome between cultivars but between time points (observed species F value = 6.159, P value = 0.0004*** and Simpson’s index F value = 2.216, P value = 0.0263*) (Suppl. Table S3). The bacterial richness of each cultivar declined significantly during the period between harvest (T2) and the dormancy break (T6) (Suppl. Figure S1B). The CAP scaling plot of six different time points during storage and the corresponding sprouts indicated a shift from harvest to sprouting (Fig. 2B). The PERMANOVA on the Bray–Curtis dissimilarity distance (9999 permutations) revealed that the microbiomes of tuber samples differed significantly between the cultivars (R2 = 0.069, P value = 0.0004***) and time points (R2 = 0.221, P value = 0.0001***) (Suppl. Table S4). The test results for cultivar (P value = 0.01**) and time point (P value = 0.01**) were confirmed by the multivariate generalized linear model for multivariate abundance data on the Bray–Curtis dissimilarity distance matrix (999 permutations) (Suppl. Table S4). To identify the bacterial taxa that changed over time during storage, differentially abundant rOTUs were calculated with the random forest function and visualized in Fig. 3 at the genus level. The relative abundance of *Staphylococcus* sp. increased during the period from harvesting (T2) to dormancy breaking (T6) from approximately 0% to 11% relative abundance. A similar result was visible for *Propionibacterium* sp. and *Acinetobacter* sp. The relative abundance of both was approximately 2% in tubers after harvesting (T2) and increased during storage to 8% and 9% in dormancy broken tubers (T6), respectively, whereas the relative abundance of the taxa *Iamia* sp. and *Nocardioides* sp. decreased from approximately 10% after harvesting to 4% and 6% in already sprouted tubers (T7), respectively (Suppl. Tables S5 and S6).

### Taxa associated with short, medium or long storage stability

To identify the bacterial taxa in the potato tuber microbiome associated with early or late potato tuber sprouting, sample data were grouped into short, medium and long storage abilities based on the individual storage time (in days from harvest until sprouting) of the samples. The data of all samples, including those of cultivar Fabiola, were considered for the following analysis (Fig. 1). After filtering for the OTUs with at least 0.01% relative abundance and presence in at least two of three replicates, 813 rOTUs were obtained. To identify rOTUs associated with short, medium or long storage stability at the beginning and end of the storage period of the potato tubers, two different analysis steps were performed. In the first step, differentially abundant rOTUs depending on storage stability (short, medium or long) and storage time points (T2, T6 and T7) were calculated with the random forest function (Suppl. Table S7). In a second step, a correlation matrix based on Spearman’s rank correlation was calculated based on the previously described factor storage stability and storage time points to identify positive or negative interactions between rOTUs (Suppl. Table S8). The same rOTUs obtained with random forest analysis, as well as with the correlation matrix, based on Spearman analysis, were filtered and named key OTUs (Suppl. Table 9). Even if both analysis steps do not provide the same evidence, they provide a meaningful indication of which OTUs are related to the factors, storage stability and storage time point. In total, we identified 24 key OTUs. The relative abundance of nine key OTUs was associated with long storage stability, meaning that the OTUs were significantly increased in samples where dormancy break (T6) and sprouting (T7) started late. These key OTUs are members of the orders *Flavobacteriales, Cytophagales*, *Sphingobacteriales, Gaiellales, Corynebacteriales*, *Caulobacterales, Methylophilales and Solirubrobacterales*. Additionally, the relative abundance of eight rOTUs was associated with short storage stability. These rOTUs are members of the orders *Enterobacteriales, Pseudomonadales, Myxococcales, Rhizobiales, Bacillales* and *Burkholderiales*. The relative abundance of seven key OTUs was associated with medium storage stability.

### Testing the effect of selected bacterial taxa on potato tuber sprouting

**Figure 4:**
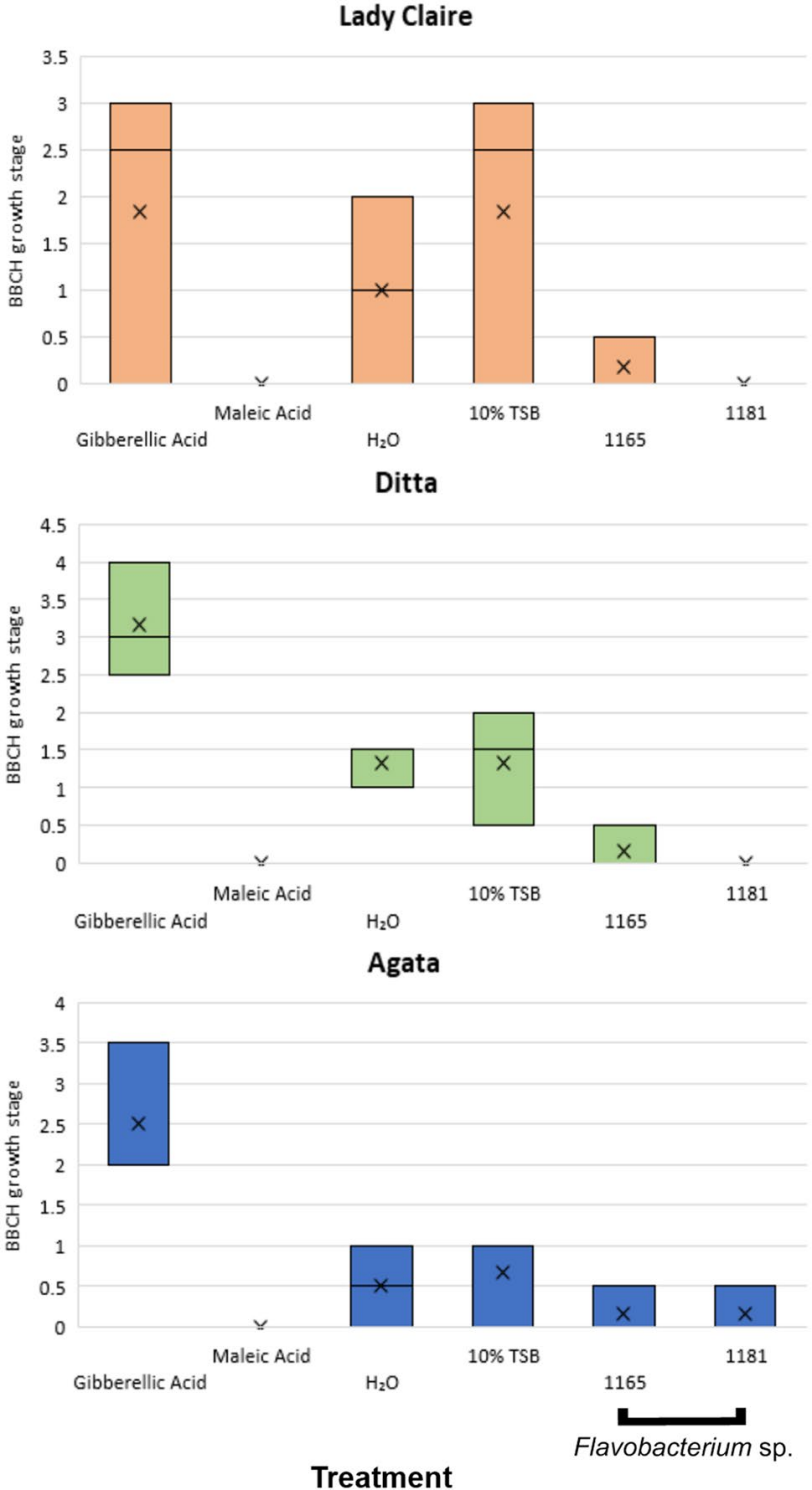
In vitro potato tuber sprouting assay results. Tuber buds of three different varieties (Lady Claire, Ditta and Agata) were treated with two different isolates of *Flavobacterium* sp. (AIT1165 and AIT1181). For evaluation, bud growth was assessed according to the first principal growth stages of the BBCH scale. Hereby, the first stage 00 is considered innate or enforced dormancy with no sprouting at all, followed by stages 01 and 02, which represent the beginning of sprouting when sprouts are visible with sizes up to < 1 mm and 2 mm, respectively. According to the BBCH scale, dormancy is broken at stage 03. When tuber buds treated with maleic hydrazine reached stage 01, the assay was ended, and the sum of all growth stages of each replicate was calculated. Afterwards, the average of two repetitions was visualized in a boxplot diagram. The boxplot diagram shows that treatments with both isolate cultures led to sprouting inhibition in comparison to the assay control. The assay control gibberellic acid represents an effective sprouting promoter, whereas maleic hydrazine was used as the negative control. Ten percent tryptic soy broth (used as the medium for culture of the isolates) and sterile water were used as neutral controls.

In the next step, we tested whether the bacterial taxa that correlated with longer storability can directly affect the sprouting of potato tubers. Therefore, we screened a collection of bacteria isolated from seed potatoes^18,20^ for isolates that are homologous in the 16S rRNA gene to the key OTUs identified in the statistical analysis. We identified two isolates that were homologous to OTU_14 (*Flavobacterium* sp.), which correlated with late sprouting. The selected isolates were tested in an in vitro sprouting assay adapted from Hartmann et al.^21^. Tuber discs treated with cultures of *Flavobacterium* sp. isolates (AIT1165 and AIT1181) resulted in sprout growth inhibition compared to control discs treated with sterile tryptic soy broth (Fig. 4). Furthermore, we observed significant individual differences in the sprouting behaviour of the tested buds. Differences in sprouting behaviour are only partly genotype-dependent, but these differences might also be due to the natural developmental variability between buds. The apical eye on a tuber usually begins to sprout first, marking the start of the apical dominance stage^22^. For the sprouting assay performed in this study, we took several buds from one tuber independently of their position.

### Screening bacterial isolates for modulation of potato tuber sprouting

The next step was to examine the common sprouting inhibition activity among potato endophytes to eliminate the possibility of a random association. Therefore, 218 randomly selected bacterial isolates from seed potatoes of the Agata, Ditta, Hermes and Lady Claire varieties, isolated elsewhere^18,20^, were tested for their effect on the potato tuber buds of the Lady Claire variety in the in vitro sprouting assay. The isolates AIT 1165 and AIT 1181 were also included in this assay and served as a benchmark. In total, 16 different bacterial isolates suppressed potato bud outgrowth (Suppl. Table 10), and the treatment of 10 of these 16 isolates was significantly different from the treatment with sterile tryptic soy broth (P value < 0.04) (Suppl. Table 11). These strains were selected for further sprouting assays with the Hermes, Tosca, Fontane and Agria varieties. Two of the 10 strains were tested, *Bacillus* sp. (AIT 1184) and *Arthrobacter* sp. (AIT 1143), resulting in sprout growth inhibition in all tested potato varieties compared to the control discs treated with sterile tryptic soy broth (Suppl. Figure S3).

## Discussion

Apart from the genotype-dependent differences in sprouting behaviour, the potato varieties used in this study exhibited differences in sprouting time, depending on the soil in which they had been grown. This finding is in line with the observations made by Austrian potato producers that batches of potatoes of the same variety, obtained from different fields, often differ in storage stability (personal communication). Possible reasons for these variations are differences in the physical and chemical parameters of the soils, weather conditions and management practices. However, in this study, potatoes were grown under identical conditions, and the statistical analysis did not reveal a correlation between the sprouting time of tubers and soil parameters. In a previous study, we have shown that the soil is the main reservoir for bacteria that colonize potato tubers, i.e., the bacterial community in tubers was recruited mainly from the soil and partly inherited from one tuber generation to the next; this recruitment process was largely independent of the plant variety^18^. Similarly, Weinert et al.^23^ showed that the bacterial community in potato tubers strongly depends on the bacterial community in soil. These studies point to the fact that differences in soil microbiota result in tubers containing different bacterial communities, which also display different activities. This could at least partly explain the differences in the observed storage stability.

From harvest to dormancy break, the bacterial communities in potato tubers underwent a strong shift, which was independent of the plant variety and the soil in which the tubers were grown. This indicates that the potato tuber microbiota is dynamic during storage. Temporal shifts in the microbial community during storage have also been reported for sugar beets, where changes in the fungal and oomycete community composition were associated with the development of soft rot. Interestingly, the shifts in the microbial community of sugar beets were also genotype independent^6^.

The relative abundances of the taxa *Propionibacterium* sp., *Staphylococcus* sp. and *Acinetobacter* sp. increased during storage, whereas *Iamia* sp. and *Nocardioides* sp. were significantly less abundant at the sprouting stage than at the harvest stage. This concurs with recently published data on the microbiota of potatoes after 5 months of storage^24^, where Actinobacteria and Firmicutes were prevalent in pulp samples. The presence of *Propionibacterium* sp. in potato tubers has also been reported previously^25^, and it has been speculated that, similar to grapevine^26^, *Propionibacterium* sp. could have been horizontally transferred from humans to potatoes during domestication. The repeated discovery of *Propionibacterium* sp. in potato tubers supports this speculation, and a closer, systemic relationship between the bacterium and potatoes seems likely.

During dormancy, potato tubers undergo structural and metabolic changes, at the end of which bud outgrowth is initiated^13^. Sugar metabolism plays a central role in this process. Sucrose, which is an important regulator of bud outgrowth^15^, is mobilized and translocated to buds, thereby decreasing in parenchyma cells. Sucrose availability is indirectly regulated by trehalose-6-phosphate (T6P) signal transduction, and a reduction in T6P increases the flux into sucrose^27^. Thus, the shifts in the bacterial community composition in potato tubers during storage could constitute the response to changes in the plant environment, especially the changes in sugar availability. In turn, tuber microbiota activity could influence sprouting initiation processes by modulating the level of sucrose or T6P. In this context, it is noteworthy that the expression of a microbial invertase or T6P-phosphatase in transgenic potatoes resulted in the inhibition of tuber sprouting^27,28^ and that the microbial conversion of sucrose into fructose and glucose in stored sugar beets is well documented^29^.

In addition to spatiotemporal changes in metabolite concentrations, plant growth hormones are important regulators of potato tuber dormancy. The initiation and duration of tuber dormancy, for example, is closely linked with the concentration of abscisic acid (ABA), which is highest immediately after harvest and declines gradually until dormancy break^15^. However, ABA is not only a plant endogenous growth hormone but is also an important signal molecule in interspecies communication, and the ability to synthesize ABA has been demonstrated for various microorganisms, including endophytic bacteria and fungi^30^. In addition, microorganisms could slow tuber sprouting by modulating the level of auxin in tubers. For example, indole-3 acetic acid (IAA) synthesized by the bacterium *Enterobacter cloacae* S11: T:07 (NRRL B-21050) and applied to cored potato eyes efficiently inhibited bud outgrowth^31^. IAA is the most common auxin in nature and controls, among other functions, cell enlargement and division, and tissue differentiation. The effect of IAA in plants is concentration dependent, and while IAA promotes cell growth up to a certain concentration, excessive concentrations have an inhibitory effect^32^. In principle, IAA synthesis is considered a mechanism by which microorganisms promote plant growth. However, if a sufficiently high concentration of IAA is achieved by microbial activity at least at the spatiotemporal level, this effect could be reversed. Furthermore, ethylene is supposed to play a role in dormancy initiation, as the concentration of ethylene temporarily increases when tubers enter dormancy^15^. In plants, 1-aminocyclopropane-1-carboxylate (ACC) serves as a precursor for the synthesis of ethylene, and many plant-associated bacteria possess an enzyme, ACC deaminase, which degrades ACC to 2-oxobutyrate and ammonia, thereby decreasing the level of ethylene in plants^33^. In terms of potato sprouting, the ACC deaminase activity of tuber-colonizing bacteria could interfere with tuber dormancy and result in bud outgrowth. Similarly, tuber-colonizing bacteria could induce premature tuber sprouting by the production of gibberellin-like substances, which has been displayed in many rhizosphere and endophytic bacteria and fungi^34,35^. Plant gibberellins are supposed to induce dormancy release and sprout growth in potato tubers, and since the concentration of gibberellins is low during dormancy, this increases before the onset of sprouting^15^.

Considering the dynamics of the bacterial communities in the potato tubers of this study, as well as the potential of microorganisms to interfere with the regulation of plant dormancy, the concept of a complex interaction between positive and negative tuber-microbiota, as well as microbiota-microbiota interactions, arises, which could result in the prolongation as well as the shortening of tuber dormancy. However, a full understanding of the role of tuber-associated microbial communities in potato sprouting behaviour would need further investigation and should also include fungi, viruses and phages.

One aim of the present study was to describe the members of the bacterial community in stored potato tubers that are associated with late sprouting and to test whether such associations could predict a direct effect on sprouting. By combining the results from the indicator species analysis, calculated with the random forest function and the correlation matrix based on Spearman’s rank correlation, we identified nine OTUs that correlated with late sprouting. For one OTU, we identified isolates in a collection of bacteria isolated from potato tubers^18,20^, which exhibited high sequence homology in the 16S rRNA gene fragment. In an in vitro assay, *Flavobacterium* sp. isolates (AIT 1165 and AIT 1181) inhibited bud outgrowth in a genotype-independent manner. The data support the hypothesis that the microbiota colonizing potato tubers may directly influence the sprouting behaviour of potatoes. However, sprout inhibiting abilities are not widespread among potato tuber endophytes, as only 1% of bacterial isolates obtained from potato tubers inhibited tuber bud outgrowth in an in vitro screening assay with four potato varieties.

The multivariant approach, used to predict associations between members of the tuber bacterial community and sprouting behaviour, proved suitable for making an educated guess as to the functional potential of microbiota in the system. In this way, it was possible to reduce the effort of microbiological screening for the desired activity in the laboratory. The integration of multiple statistical methods has also been used to identify taxa within soil microbial communities involved in pine needle litter decomposition^36^. However, the potato tubers used in the present study were obtained from a long-term experiment, which was designed to reduce natural variations as much as possible to carve out the effect of the bacterial communities of tubers in relation to sprouting behaviour^18^. We do not know to what extent the statistical approach used in this study is applicable in environmental studies with the plurality of unpredictable influencing factors. The present study, nevertheless, is a good example indicating that microbial ecology studies and the obtained community understanding can provide a basis for the identification of microbial functions for biotechnological applications.

## Materials and methods

### Cultivation of potato tubers

The cultivation of potato tubers used in this study has recently been described in Buchholz et al., and an overview of the plant experiments is provided in Supplementary Figure S2. Seed potatoes of four different cultivars of *Solanum tuberosum* L. (Hermes, Lady Claire, Fabiola and Agata) were sown in 6 L pots filled with commercial potting soil (Profi Substrat, Einheitserde special, Sinntal-Altengronau, Germany), grown in the greenhouse and fertilized twice with NPK + trace elements (Compo Austria GmbH, Vienna, Austria). During the day, the temperature of the greenhouse was set to 22 °C with 50% humidity, and at night the settings were 21 °C with 35% humidity. Potato plants were grown in separate pots with individual saucers to avoid cross contamination. All plants were watered from above with a volume of one to 1.5 L every second to third day and kept sufficiently moist during cultivation. Tubers (later named first generation tubers) were harvested when they reached growth stage 909 according to the BBCH scale^37^ (https://www.julius-kuehn.de/publikationsreihen-des-jki/bbch-skala/). This stage is defined as the senescence stage. Here, leaves and stems are already dead, and stems are also bleached and dry^37^.

In May 2016, tubers were planted in 10 L pots filled with commercial potting soil (Profi Substrat, Einheitserde special, Sinntal-Altengronau, Germany) and four different farmland soils from different regions of Lower Austria. Farmland soils were obtained from fields in Karnabrunn, Tulln and Kettlasbrunn, whereby two different types of soil were collected in Kettlasbrunn. One of the soils from Kettlasbrunn (Kettlasbrunn A) was overfertilized with manure (personal communication with the farmer). The soil characteristics are summarized in Suppl. Table S1. Before sowing, tubers were incubated in 2 ppm gibberellic acid (Sigma-Aldrich, Vienna, Austria) for 20 min and subsequently dried to promote rapid germination (adapted from^21^). Plant cultivation was performed as described above. Tubers (later named second generation tubers) were harvested at growth stage 909 of the BBCH scale^37^.

### Storage of potato tubers

After harvesting, second generation tubers were stored in darkness at 8–10 °C in the refrigerator (Liebherr Profi Line UKS 3602, Kirchdorf, Germany). Potato tubers belonging to the same variety/soil type combination (e.g., Lady Claire grown in Karnabrunn) were collected in one paper bag. Tubers were sampled immediately after harvesting and after 2, 5 and 10 weeks of storage independent of the variety and soil type in which tubers were cultivated. In the first 10 weeks after harvesting, potato tubers were in a physiological stage of deep dormancy. After that, tubers were checked weekly until all tubers belonging to a variety/soil type combination reached the end of dormancy (BBCH stage 003 characterized as the end of dormancy: sprouts 2–3 mm) and the sprouting stage (BBCH stage 005 characterized as the beginning of root formation), and then the samples were collected^37^. These sampling timepoints were different for all variety/soil type combinations. At each stage, three tubers of each variety/soil type combination were sampled. Overall, the different sampling time points were reached when at least the apical eye of all three replicate tubers reached the desired BBCH growth stage. An overview of the sampling is shown in Fig. 1.

### Sampling of potato tubers, DNA isolation and soil analysis

For sampling, three buds per tuber (biological replicate) were taken, and 5-mm-thick bud discs were cut from the corky epidermis, the cortex, the outer medulla and the inner medulla with an estimated size of 9–10 g. Each bud disc was placed into a sterile pulsifier bag together with 6 mL of sterile 10% tryptic soy broth (Sigma-Aldrich, Austria) and homogenized three times for 15 s with a Pulsifier (Microgen Bioproducts, Surrey, UK). To obtain a representative overview of the microbial community of each potato tuber, 1 mL of each sample taken from the corky epidermis, cortex, outer and inner medulla were mixed together, and two mL of the pools were used for DNA isolation with the FastDNA SPIN Kit for Soil (MP Biomedicals, Solon, OH, USA) as described elsewhere^18^. Additionally, 500 g of bulk soil of each farmland soil type and potting soil were sampled and analysed for their chemical parameters and clay contents by the Austrian Agency for Health and Food Safety (AGES GmbH).

### 16S rRNA gene amplicon sequencing and sequencing data processing and statistics

The bacterial communities in potato tubers were assessed by sequencing amplicons of the V5–V7 region of the 16S rRNA gene as described elsewhere^38,39^. In brief, 16S rRNA gene amplicon libraries were generated using two rounds of PCR amplification. The first PCR was performed with the PCR primers 799f. (5′-AACMGGATTAGATACCCKG-3′) and 1175r (5′-ACGTCRTCCCCDCCTTCCT-3′)^40^. The bacterial 16S rRNA gene amplicons (approximately 400 bp) were separated from mitochondrial 18S rRNA gene amplicons by electrophoresis and excised from the gel. The second PCR round was performed with the primers 799f. and 1175r, which carry specific indices (Suppl. Table S12), provided by LGC (LGC Genomics, Berlin, Germany). Illumina-adapter ligation and sequencing were performed using 2 × 250 bp MiSeq v2 sequencing (Illumina Inc. San Diego, CA, USA) at LGC Genomics (LGC Genomics, Berlin, Germany).

Data processing and statistical analysis were performed as described in a previous paper^18^, and detailed descriptions are provided in “Supplementary Methods”.

### Nucleotide sequence accession numbers

Sequence data are available in the NCBI SRA database under the accession number SRR8432951 and the BioProject number PRJNA513967.

### Correlation networks and selection of functional OTUs

We employed correlation network analysis to identify OTUs with potentially positive or negative effects on the storage time of potato tubers^41^. Prior to correlation analysis, we filtered the OTU table by removing OTUs with > 66.66% ties (i.e., OTUs that had the same number of reads in more than two-thirds of the samples, usually zero reads) and OTUs that constituted less than 0.01% of the total reads. The resulting OTU table including an additional column containing the number of days until tubers reached BBCH stage 909^37^, which is a proxy for the suitability of long storage, was used to calculate a correlation matrix based on Spearman’s rank correlation. Correlations with a correlation coefficient, r, < 0.5 and with significance corresponding to p > 0.001 were set to zero to restrict network analysis to strong and highly significant correlations.

### Isolation and characterization of bacteria from potato tubers

Isolation and identification of the bacteria colonizing the potato tubers was performed in a parallel study^20^. In brief, potato sample preparation was performed as described above. One hundred microlitres of the homogenate was diluted 1:10 and 1:100. Ten microlitres of these dilutions were plated on 10% tryptic soy agar (Merck, KGaG, Darmstadt, Germany) and incubated at 28 °C. Phenotypically different colonies were picked and reinoculated on fresh plates four times to obtain pure cultures. For identification of the isolates, fast DNA extraction was performed with InstaGene Matrix Solution (BioRad Laboratories, Inc., Vienna, Austria), and the 16S rRNA gene region was amplified as described elsewhere^20^ using primers 8f. (5′-AGAATTTGATCCTGGCTCAG-3′) and 1520r (5′-AAGGAGGTGATCCAGCCGCA-3′)^42^. Purified fragments were sequenced at GATC Biotech (Konstanz, Germany) with the primer 1492r (5′-CGGTTACCTTGTTACGACTT-3′) slightly modified from Bayers et al.^43^. Sequence data are available in the NCBI database and GenBank under the accession numbers MT655318—MT655535.

Sequences of OTUs identified in the statistical analysis were aligned to the 16S rRNA gene fragments of isolates using the Multiple sequence alignment tool^44^, and isolates that were homologous to the OTUs in question were selected for further testing.

### Sprouting assay

An in vitro tuber-sprouting assay was adapted from Hartmann et al.^21^. Potato tubers were washed with tap water to remove residues of soil and were dried for 16 h. Afterwards, 5 mm discs containing one bud each were cut with an apple corer. Before starting the assay, bud discs of one potato variety were mixed with each other to ensure even distribution across treatments. Discs were incubated in 2 ppm gibberellic acid (Merck KGaA, Darmstadt, Germany), maleic hydrazide (Merck KGaA, Darmstadt, Germany) with a concentration of 4 g/L, sterile water, sterile 10% TSB solution or different bacterial cultures each with a density of 0.1 at OD 600 (between 10^7^ to 10^8^ cfu/ml, depending on the taxon). A maximum of six discs were incubated in 25 mL of each treatment solution for 15 min on an orbital shaker (RS-TR05, Phoenix Instruments, Garbsen, Germany). Then, each treated disc was placed into one well of a 12-well plate (Sarstedt, Nümbrecht, Germany). Twelve-well plates were filled with two mL of water agar. Afterwards, plates were sealed with parafilm and stored at 8 °C. The sprouting behaviour of tuber discs was evaluated every 24 h until discs treated with maleic hydrazine started sprouting and reached stage 01 of the BBCH scale. This stage is defined as the beginning of potato tuber sprouting or the beginning of seed imbibition, and sprouts are visible at approximately < 1 mm^37^. After that, assay results were analysed in the R v3.6.2 software environment^45^. We evaluated statistical significance at α = 0.05. The effect of the individual treatments on each potato bud was assessed by fitting a linear model. This analysis was followed by an analysis of variance (ANOVA) and a post hoc study to test which bacterial treatment led to significant sprouting inhibition in comparison to treatment with the control, 10% tryptic soy broth. This was realized by employing an estimated marginal mean (EMM) approach^46^.

## Supplementary Information

Supplementary Information.

## Data Availability

The sequencing datasets generated during and analysed during the current study are available in the NCBI SRA database under the accession number SRR8432951 and the BioProject number PRJNA513967. Sequence data from Sanger sequencing are available from the NCBI database and GenBank under the accession numbers MT655318–MT655535.
