# Supplementary material for: 16S rRNA gene-based microbiome analysis identifies candidate bacterial strains that increase the storage time of potato tubers
Source: Sci Rep. 2021 Feb 4;11:3146. doi: 10.1038/s41598-021-82181-9 (PMC7862659; doi:10.1038/s41598-021-82181-9)
Supplement: Supplementary file 1 — Supplementary Information. [file 41598_2021_82181_MOESM1_ESM.docx]

Supplementary information for

**16S rRNA gene-based microbiome analysis identifies candidate bacterial strains that increase the storage time of potato tubers**

Franziska Buchholz, Robert Junker, Abdul Samad, Livio Antonielli, Nataša Sarić, Tanja Kostić, Angela Sessitsch and Birgit Mitter

Author for correspondence:

Birgit Mitter, email: birgit.mitter@ait.ac.at

**This file includes:**

Supplementary Tables 1-12

Supplementary Figures 1-3

Supplementary Methods

**Supplementary Table S1: Storage time of potato tuber varieties and their corresponding soil type.** The table shows the individual storage time of tubers in days after harvesting. Additionally, the calculated average (d) for each soil type/variety combination is exposed. Sample of T7 Karnabrunn/Fabiola is missing because not enough tubers were left after harvesting.

| **Soil type** | **Variety** | **Days after harvesting [d]** | **Average [d]** |
| --- | --- | --- | --- |
| potting soil | Agata | 116 | 146 |
|  | Hermes | 161 |  |
|  | Lady Claire | 154 |  |
|  | Fabiola | 151 |  |
| Tulln | Agata | 116 | 143 |
|  | Hermes | 158 |  |
|  | Lady Claire | 138 |  |
|  | Fabiola | 159 |  |
| Kettlasbrunn A | Agata | 131 | 145 |
|  | Hermes | 168 |  |
|  | Lady Claire | 147 |  |
|  | Fabiola | 135 |  |
| Kettlasbrunn B | Agata | 125 | 143 |
|  | Hermes | 158 |  |
|  | Lady Claire | 147 |  |
| Karnabrunn | Agata | 131 | 155 |
|  | Hermes | 164 |  |
|  | Lady Claire | 155 |  |
|  | Fabiola | 169 |  |

**Supplementary Table S2: Chemical soil properties of each farmland soil type and potting soil.** Soil parameter were measured by the Austrian Agency for Heath and Food Safety (AGES GmbH).

| **Sample** | **pH-value: CaCl2** | **P [mg/kg]** | **K [mg/kg]** | **N [%]** | **Clay content [%]** | **Ca [cmolc/kg]** | **Mg [cmolc/kg]** | **K [cmolc/kg]** | **Na [cmolc/kg]** | **Al [cmolc/kg]** | **Mn [cmolc/kg]** | **Exchange capacity [cmolc/kg]** |
| --- | --- | --- | --- | --- | --- | --- | --- | --- | --- | --- | --- | --- |
| Karnabrunn | 7.4 | 223.00 | 293.00 | 0.17 | 22.00 | 18.84 | 1.10 | 0.95 | 0.04 | 0.06 | 0.01 | 20.91 |
| Kettlasbrunn A | 7.5 | 110.00 | 257.00 | 0.20 | 26.00 | 23.78 | 1.38 | 0.79 | 0.07 | 0.06 | 0.01 | 26.02 |
| Kettlasbrunn B | 7.5 | 100.00 | 287.00 | 0.17 | 20.00 | 22.12 | 1.28 | 0.90 | 0.07 | 0.06 | 0.01 | 24.36 |
| Potting Soil | 6.5 | n.d. | n.d. | 0.69 | 0.00 | 75.73 | 11.3 | 4.67 | 0.52 | 0.06 | 0.07 | 92.28 |
| Tulln | 7.6 | 28.00 | 66.00 | 0.15 | 26.00 | 21.84 | 1.98 | 0.24 | 0.08 | 0.06 | 0.01 | 24.24 |

**Supplementary Table S3: Statistical analysis of alpha diversity values measured by bacterial richness and Simpson index.** Data were accomplished with permutation ANOVA and pairwise comparison permutation t-test between the following factors: time point, soil type and/or cultivar.

**Dataset: potato tuber of the varieties Agata, Lady Claire and Hermes cultivated in five different soil types (T2), stored until dormancy break (T6) and until sprouting (T7) as well as samples of the sprouts (T7_Sprouts):**

| **observed species** | | | | | |  |
| --- | --- | --- | --- | --- | --- | --- |
| **Permutation Analysis of Variance Table** | | |  |  |  |  |
| 9999 permutations | |  |  |  |  |  |
|  |  |  |  |  |  |  |
|  | Sum Sq | Df | Mean Sq | F value | Pr(>F) |  |
| **Cultivar** | 90301 | 2 | 45151 | 3.9633 | 0.0211 | * |
| Residuals | 1982237 | 174 | 11392 |  |  |  |
|  |  |  |  |  |  |  |
|  | Sum Sq | Df | Mean Sq | F value | Pr(>F) |  |
| **Timepoint** | 158079 | 3 | 52693 | 4.7616 | 0.0021 | ** |
| Residuals | 1914459 | 173 | 11066 |  |  |  |
|  |  |  |  |  |  |  |
|  | Sum Sq | Df | Mean Sq | F value | Pr(>F) |  |
| **Soil_type** | 167415 | 4 | 41854 | 3.7787 | 0.0057 | ** |
| Residuals | 1905123 | 172 | 11076 |  |  |  |
|  |  |  |  |  |  |  |
| Signif. codes: 0 ‘***’ 0.001 ‘**’ 0.01 ‘*’ 0.05 ‘.’ 0.1 ‘ ’ 1 | | | |  |  |  |

| **Pairwise comparisons using permutation t tests** | | | | | | | | |  | | |  | |  | | |  |  |  |
| --- | --- | --- | --- | --- | --- | --- | --- | --- | --- | --- | --- | --- | --- | --- | --- | --- | --- | --- | --- |
| 9999 permutations | | | | |  | | | |  | | |  | |  | | |  |  |  |
|  |  | | |  |  |  |  |  |  |  |  |  |  |  |  |  |  |  |  |
|  | | | Agata | | Hermes | | | |  | | |  | |  | | |  |  |  |
| Hermes | | | 0.202 | | - | | | |  | | |  | |  | | |  |  |  |
| Lady Claire | | | 0.003 | | 0.22 | | | |  | | |  | |  | | |  |  |  |
|  |  | | |  |  |  |  |  |  |  |  |  |  |  |  |  |  |  |  |
|  | | | T2 | | T6 | | | | T7 | | |  | |  | | |  |  |  |
| T6 | | | 0.017 | | - | | | | - | | |  | |  | | |  |  |  |
| T7 | | | 0.651 | | 0.017 | | | | - | | |  | |  | | |  |  |  |
| T7_Sprouts | | | 0.019 | | 0.600 | | | | 0.017 | | |  | |  | | |  |  |  |
|  | | |  | |  | | | |  | | |  | |  | | |  |  |  |
|  | | | Karnabrunn | | Kettlasbrunn A | | | | Kettlasbrunn B | | | Potting soil | |  | | |  |  |  |
| Kettlasbrunn A | | | 0.751 | | - | | | | - | | | - | |  | | |  |  |  |
| Kettlasbrunn B | | | 0.268 | | 0.200 | | | | - | | | - | |  | | |  |  |  |
| Potting soil | | | 0.126 | | 0.078 | | | | 0.370 | | | - | |  | | |  |  |  |
| Tulln | | | 0.024 | | 0.006 | | | | 0.024 | | | 0.292 | |  | | |  |  |  |
|  | | |  | |  | | | |  | | |  | |  | | |  |  |  |
| P value adjustment method: fdr | | | | | | | | |  | | |  | |  | | |  |  |  |
| **Simpson Index** | | | | | | | | | | | | | | | | | |  |  |
| **Permutation Analysis of Variance Table** | | | | | | | |  | | |  | | |  | |  | |  |  |
| 9999 permutations | | | | | |  | |  | | |  | | |  | |  | |  |  |
|  | |  | | | |  | |  | | |  | | |  | |  | |  |  |
|  | | Sum Sq | | | | Df | | Mean Sq | | | F value | | | Pr(>F) | |  | |  |  |
| **Cultivar** | | 0.07658 | | | | 2 | | 0.038288 | | | 4.5941 | | | 0.0104 | | * | |  |  |
| Residuals | | 1.45012 | | | | 174 | | 0.008334 | | |  | | |  | |  | |  |  |
|  | |  | | | |  | |  | | |  | | |  | |  | |  |  |
|  | | Sum Sq | | | | Df | | Mean Sq | | | F value | | | Pr(>F) | |  | |  |  |
| **Timepoint** | | 0.1598 | | | | 3 | | 0.053266 | | | 6.7415 | | | 4.00E-04 | | *** | |  |  |
| Residuals | | 1.3669 | | | | 173 | | 0.007901 | | |  | | |  | |  | |  |  |
|  | |  | | | |  | |  | | |  | | |  | |  | |  |  |
|  | | Sum Sq | | | | Df | | Mean Sq | | | F value | | | Pr(>F) | |  | |  |  |
| **Soil_type** | | 0.16252 | | | | 4 | | 0.040630 | | | 5.1228 | | | 8.00E-04 | | *** | |  |  |
| Residuals | | 1.36418 | | | | 172 | | 0.007931 | | |  | | |  | |  | |  |  |
|  | |  | | | |  | |  | | |  | | |  | |  | |  |  |
| Signif. codes: 0 ‘***’ 0.001 ‘**’ 0.01 ‘*’ 0.05 ‘.’ 0.1 ‘ ’ 1 | | | | | | | | | | |  | | |  | |  | |  |  |
| Pairwise comparisons using permutation t tests | | | | | | | | | |  | | |  | |  | | | |  |
| 9999 permutations | | | | | | |  | | |  | | |  | |  | | | |  |
|  | | |  | | | |  | | |  | | |  | |  | | | |  |
|  | | | Agata | | | | Hermes | | |  | | |  | |  | | | |  |
| Hermes | | | 0.1428 | | | | - | | |  | | |  | |  | | | |  |
| Lady Claire | | | 0.0048 | | | | 0.1428 | | |  | | |  | |  | | | |  |
|  | | |  | | | |  | | |  | | |  | |  | | | |  |
|  | | | T2 | | | | T6 | | | T7 | | |  | |  | | | |  |
| T6 | | | 0.1506 | | | | - | | | - | | |  | |  | | | |  |
| T7 | | | 0.1620 | | | | 0.8588 | | | - | | |  | |  | | | |  |
| T7_Sprouts | | | 0.0500 | | | | 0.0048 | | | 0.0060 | | |  | |  | | | |  |
|  | | |  | | | |  | | |  | | |  | |  | | | |  |
|  | | | Karnabrunn | | | | Kettlasbrunn A | | | Kettlasbrunn B | | | Potting soil | |  | | | |  |
| Kettlasbrunn A | | | 0.1930 | | | | - | | | - | | | - | |  | | | |  |
| Kettlasbrunn B | | | 0.2463 | | | | 0.8076 | | | - | | | - | |  | | | |  |
| Potting soil | | | 0.0005 | | | | 0.0005 | | | 0.0005 | | | - | |  | | | |  |
| Tulln | | | 0.1930 | | | | 0.8076 | | | 0.8048 | | | 0.0005 | |  | | | |  |
|  | | |  | | | |  | | |  | | |  | |  | | | |  |
| P value adjustment method: fdr | | | | | | | | | |  | | |  | |  | | | |  |

**Dataset: potato tubers of the varieties Agata, Lady Claire and Hermes grown in potting soil at six different timepoints: T2 (harvest), T3 (two weeks after harvesting), T4 (five weeks after harvesting), T5 (ten weeks after harvesting), T6 (dormancy break), T7 (sprouting) and T7_Sprouts (corresponding sprouts)**

| **observed species** | | | | | | | | | | | |  | |
| --- | --- | --- | --- | --- | --- | --- | --- | --- | --- | --- | --- | --- | --- |
| **Permutation Analysis of Variance Table** | | | | | |  | |  |  | | |  | |
| 9999 permutations | | | |  | |  | |  |  | | |  | |
|  | |  | |  | |  | |  |  | | |  | |
|  | | Sum Sq | | Df | | Mean Sq | | F value | Pr(>F) | | |  | |
| Cultivar | | 22055 | | 2 | | 11028 | | 1.5783 | 0.2168 | | |  | |
| Residuals | | 405244 | | 58 | | 6987 | |  |  | | |  | |
|  | |  | |  | |  | |  |  | | |  | |
|  | | Sum Sq | | Df | | Mean Sq | | F value | Pr(>F) | | |  | |
| Timepoint | | 173607 | | 6 | | 28935 | | 6.1589 | 1.00E-04 | | | *** | |
| Residuals | | 253692 | | 54 | | 4698 | |  |  | | |  | |
|  | |  | |  | |  | |  |  | | |  | |
| Signif. codes: 0 ‘***’ 0.001 ‘**’ 0.01 ‘*’ 0.05 ‘.’ 0.1 ‘ ’ 1 | | | | | | | |  |  | | |  | |
|  | |  | |  | |  | |  |  | | |  | |
| **Pairwise comparisons using permutation t tests** | | | | | |  | |  |  | | |  | |
| 9999 permutations | | | |  | |  | |  |  | | |  | |
|  | |  | |  | |  | |  |  | | |  | |
|  | | Agata | | Hermes | |  | |  |  | | |  | |
| Hermes | | 0.28 | | - | |  | |  |  | | |  | |
| Lady Claire | | 0.44 | | 0.44 | |  | |  |  | | |  | |
|  | |  | |  | |  | |  |  | | |  | |
|  | | T2 | | T3 | | T4 | | T5 | T6 | | | T7 | |
| T3 | | 0.5339 | | - | | - | | - | - | | | - | |
| T4 | | 0.0109 | | 0.0651 | | - | | - | - | | | - | |
| T5 | | 0.0028 | | 0.0168 | | 0.7272 | | - | - | | | - | |
| T6 | | 0.0028 | | 0.0042 | | 0.4434 | | 0.0651 | - | | | - | |
| T7 | | 0.2178 | | 0.7211 | | 0.0844 | | 0.0168 | 0.0028 | | | - | |
| T7_Sprouts | | 0.2178 | | 0.4946 | | 0.309 | | 0.3116 | 0.0641 | | | 0.6435 | |
|  | |  | |  | |  | |  |  | | |  | |
| P value adjustment method: fdr | | | | | |  | |  |  | | |  | |
| **Simpson Index** | | | | | | | | | | | | | |
| **Permutation Analysis of Variance Table** | | | | | |  | | |  |  | |  | |
| 9999 permutations | | | |  | |  | | |  |  | |  | |
|  | |  | |  | |  | | |  |  | |  | |
|  | | Sum Sq | | Df | | Mean Sq | | | F value | Pr(>F) | |  | |
| **Cultivar** | | 0.001164 | | 2 | | 0.00058223 | | | 0.8513 | 0.4895 | |  | |
| Residuals | | 0.039667 | | 58 | | 0.00068391 | | |  |  | |  | |
|  | |  | |  | |  | | |  |  | |  | |
|  | | Sum Sq | | Df | | Mean Sq | | | F value | Pr(>F) | |  | |
| **Timepoint** | | 0.008067 | | 6 | | 0.00134451 | | | 2.2159 | 0.0263 | | * | |
| Residuals | | 0.032764 | | 54 | | 0.00060675 | | |  |  | |  | |
|  | |  | |  | |  | | |  |  | |  | |
| Signif. codes: 0 ‘***’ 0.001 ‘**’ 0.01 ‘*’ 0.05 ‘.’ 0.1 ‘ ’ 1 | | | | | | | | |  |  | |  | |
|  | |  | |  | |  | | |  |  | |  | |
| **Pairwise comparisons using permutation t tests** | | | | | |  | | |  |  | |  | |
| 9999 permutations | | | |  | |  | | |  |  | |  | |
|  | |  | |  | |  | | |  |  | |  | |
|  | | Agata | | Hermes | |  | | |  |  | |  | |
| Hermes | | 0.64 | | - | |  | | |  |  | |  | |
| Lady Claire | | 0.64 | | 0.64 | |  | | |  |  | |  | |
|  | |  | |  | |  | | |  |  | |  | |
|  | | T2 | | T3 | | T4 | | | T5 | T6 | | T7 | |
| T3 | | 0.71 | | - | | - | | | - | - | | - | |
| T4 | | 0.71 | | 0.92 | | - | | | - | - | | - | |
| T5 | | 0.38 | | 0.41 | | 0.41 | | | - | - | | - | |
| T6 | | 0.41 | | 0.64 | | 0.51 | | | 0.51 | - | | - | |
| T7 | | 0.71 | | 0.92 | | 0.92 | | | 0.41 | 0.56 | | - | |
| T7_Sprouts | | 0.71 | | 0.51 | | 0.51 | | | 0.29 | 0.29 | | 0.51 | |
|  | |  | |  | |  | | |  |  | |  | |
| P value adjustment method: fdr | | | | | |  | | |  |  | |  | |

**Supplementary Table S4:** **Statistical analysis of beta diversity.** Data were calculated with PERMANOVA, permutation CAP test and multivariate generalized linear model based on the Bray- Curtis dissimilarity matrix.

**Dataset: potato tuber of the varieties Agata, Lady Claire and Hermes cultivated in five different soil types (T2), stored until dormancy break (T6) and until sprouting (T7) as well as samples of the sprouts (T7_Sprouts):**

| **Permutation test for homogeneity of multivariate dispersions** | | | | | | | |  | |  |  |
| --- | --- | --- | --- | --- | --- | --- | --- | --- | --- | --- | --- |
| Number of permutations: 999 | | |  | |  | |  |  | |  |  |
| **Cultivar** |  | |  | |  | |  |  | |  |  |
|  | Df | | Sums Sq | | Mean Sq | | F.Model | N.Perm | | Pr(>F) |  |
| Groups | 2 | | 0.01818 | | 0.0090912 | | 0.6853 | 999 | | 0.535 | . |
| Residuals | 174 | | 230.815 | | 0.0132652 | |  |  | |  |  |
|  |  | |  | |  | |  |  | |  |  |
| **Timepoint** |  | |  | |  | |  |  | |  |  |
|  | Df | | Sums Sq | | Mean Sq | | F.Model | N.Perm | | Pr(>F) |  |
| Groups | 3 | | 0.09601 | | 0.032004 | | 2.242 | 999 | | 0.075 | . |
| Residuals | 173 | | 246.959 | | 0.014275 | |  |  | |  |  |
|  |  | |  | |  | |  |  | |  |  |
| **Soil type** |  | |  | |  | |  |  | |  |  |
|  | Df | | Sums Sq | | Mean Sq | | F.Model | N.Perm | | Pr(>F) |  |
| Groups | 4 | | 0.16397 | | 0.040992 | | 4.6189 | 999 | | 0.004 | ** |
| Residuals | 172 | | 152.648 | | 0.008875 | |  |  | |  |  |
| --- |  | |  | |  | |  |  | |  |  |
| Signif. codes: 0 ‘***’ 0.001 ‘**’ 0.01 ‘*’ 0.05 ‘.’ 0.1 ‘ ’ 1 | | | | | | |  |  | |  |  |
| **Pairwise comparisons:** | | | |  | |  | | |  |  |  |
| (Observed p-value below diagonal, permuted p-value above diagonal) | | | | | | | | |  |  |  |
|  | | Agata | | Hermes | | Lady | | |  |  |  |
| Agata | | 0.90600 | | 0.2890 | |  | | |  |  |  |
| Hermes | | 0.91523 | |  | | 0.365 | | |  |  |  |
| Lady Claire | | 0.26368 | | 0.3345 | |  | | |  |  |  |

|  | T2 | T6 | T7 | T7_Sprouts |
| --- | --- | --- | --- | --- |
| T2 |  | 0.078000 | 0.191000 | 0.010 |
| T6 | 0.071677 |  | 0.720000 | 0.405 |
| T7 | 0.174353 | 0.725135 |  | 0.264 |
| T7_Sprouts | 0.012284 | 0.417235 | 0.268218 |  |

|  | Karnabrunn | Kettlasbrunn A | Kettlasbrunn B | Substrat | Tulln |
| --- | --- | --- | --- | --- | --- |
| Karnabrunn | 1.00E+03 | 2.00E+02 | 5.70E+03 | 0.216 |  |
| Kettlasbrunn A | 9.73E+02 |  |  | 2.59E+03 | 0.569 |
| Kettlasbrunn B | 2.56E+02 | 2.53E+03 |  |  | 0.139 |
| Substrat | 5.34E+03 | 7.73E-01 | 2.41E-02 |  | 0.003 |
| Tulln |  | 2.27E+03 | 5.72E+03 | 1.38E+03 | 4.74E+01 |

| **permutation MANOVA on a distance matrix** | | |  |  |  |  |  |
| --- | --- | --- | --- | --- | --- | --- | --- |
|  |  |  |  |  |  |  |  |
| Number of permutations: 9999 | |  |  |  |  |  |  |
|  | Df | SumsOfSqs | MeanSqs | F.Model | R2 | Pr(>F) |  |
| Cultivar | 2 | 0.5082 | 0.25411 | 2.37910 | 0.02662 | 0.0021 | ** |
| Residuals | 174 | 18.5841 | 0.10680 | 0.97338 |  |  |  |
| Total | 176 | 19.0923 |  | 1.00000 |  |  |  |
|  |  |  |  |  |  |  |  |
|  | Df | SumsOfSqs | MeanSqs | F.Model | R2 | Pr(>F) |  |
| Timepoint | 3 | 2.1517 | 0.71722 | 7.3243 | 0.1127 | 1.00E-04 | *** |
| Residuals | 173 | 16.9407 | 0.09792 | 0.8873 |  |  |  |
| Total | 176 | 19.0923 |  | 1.0000 |  |  |  |
|  |  |  |  |  |  |  |  |
|  | Df | SumsOfSqs | MeanSqs | F.Model | R2 | Pr(>F) |  |
| Soil_type | 4 | 4.8755 | 1.21889 | 14.7470 | 0.25537 | 1.00E-04 | *** |
| Residuals | 172 | 14.2168 | 0.08266 | 0.74463 |  |  |  |
| Total | 176 | 19.0923 |  | 1.00000 |  |  |  |
|  |  |  |  |  |  |  |  |
| Signif. codes: 0 ‘***’ 0.001 ‘**’ 0.01 ‘*’ 0.05 ‘.’ 0.1 ‘ ’ 1 | | |  |  |  |  |  |

| **Pairwise comparisons using permutation MANOVAs on a distance matrix** | | | |  |
| --- | --- | --- | --- | --- |
| Number of permutations: 9999 | |  |  |  |
|  |  |  |  |  |
|  | Agata | Hermes |  |  |
| Hermes | 0.013 | - |  |  |
| Lady Claire | 0.013 | 0.079 |  |  |
|  |  |  |  |  |
|  | T2 | T6 | T7 |  |
| T6 | 0.0002 | - | - |  |
| T7 | 0.0002 | 0.0929 | - |  |
| T7_Sprouts | 0.0002 | 0.0193 | 0.0502 |  |
|  |  |  |  |  |
|  | Karnabrunn | Kettlasbrunn A | Kettlasbrunn B | Potting soil |
| Kettlasbrunn A | 0.00180 | - | - | - |
| Kettlasbrunn B | 0.00022 | 0.00012 | - | - |
| Potting soil | 0.00012 | 0.00012 | 0.00012 | - |
| Tulln | 0.00012 | 0.00012 | 0.00012 | 0.00012 |
|  |  |  |  |  |
| P value adjustment method: fdr | |  |  |  |

| **permutation CAP test on Bray Curtis dissimilarity matrix** | | |  |  |  |
| --- | --- | --- | --- | --- | --- |
| Number of permutations: 999 | |  |  |  |  |
|  |  |  |  |  |  |
|  | Df | SumOfSqs | F | Pr(>F) |  |
| Timepoint | 3 | 2.1520 | 12.2378 | 0.001 | *** |
| Cultivar | 2 | 0.5036 | 4.2961 | 0.001 | *** |
| Soil_type | 4 | 4.8855 | 20.8373 | 0.001 | *** |
| Timepoint:Cultivar | 6 | 0.6556 | 1.8640 | 0.002 | ** |
| Timepoint:Soil_type | 12 | 0.9948 | 1.4143 | 0.004 | ** |
| Cultivar:Soil_type | 8 | 1.1545 | 2.4620 | 0.001 | *** |
| Timepoint:Cultivar:Soil_type | 24 | 1.9608 | 1.3939 | 0.001 | *** |
| Residual | 117 | 6.8579 |  |  |  |
|  |  |  |  |  |  |
| Signif. codes: 0 ‘***’ 0.001 ‘**’ 0.01 ‘*’ 0.05 ‘.’ 0.1 ‘ ’ 1 | | |  |  |  |

| **multivariate GLM** | |  |  |  |  |
| --- | --- | --- | --- | --- | --- |
|  | Res.Df | Df.diff | Dev | Pr(>Dev) |  |
| (Intercept) | 176 |  |  |  |  |
| **Cultivar** | 174 | 2 | 4929 | 0.01 | ** |
|  |  |  |  |  |  |
|  | Res.Df | Df.diff | Dev | Pr(>Dev) |  |
| (Intercept) | 176 |  |  |  |  |
| **Timepoint** | 173 | 3 | 21396 | 0.01 | ** |
|  |  |  |  |  |  |
|  | Res.Df | Df.diff | Dev | Pr(>Dev) |  |
| (Intercept) | 176 |  |  |  |  |
| **Soil_type** | 172 | 4 | 45235 | 0.01 | ** |
|  |  |  |  |  |  |
| Signif. codes: 0 ‘***’ 0.001 ‘**’ 0.01 ‘*’ 0.05 ‘.’ 0.1 ‘ ’ 1 | | | |  |  |

**Dataset: Potato tubers of the varieties Agata, Lady Claire and Hermes grown in potting soil at six different timepoints: T2 (harvest), T3 (two weeks after harvesting), T4 (five weeks after harvesting), T5 (ten weeks after harvesting), T6 (dormancy break), T7 (sprouting) and T7_Sprouts (corresponding sprouts):**

| **Permutation test for homogeneity of multivariate dispersions** | | | | |  |  |  |
| --- | --- | --- | --- | --- | --- | --- | --- |
| Number of permutations: 999 | |  |  |  |  |  |  |
|  |  |  |  |  |  |  |  |
| **Cultivar** |  |  |  |  |  |  |  |
|  | Df | Sums Sq | Mean Sq | F.Model | N.Perm | Pr(>F) |  |
| Groups | 2 | 0.01480 | 0.0074013 | 1.2942 | 999 | 0.302 |  |
| Residuals | 58 | 0.33168 | 0.0057187 |  |  |  |  |
|  |  |  |  |  |  |  |  |
| **Timepoint** |  |  |  |  |  |  |  |
|  | Df | Sums Sq | Mean Sq | F.Model | R2 | Pr(>F) |  |
| Groups | 6 | 0.085964 | 0.0143273 | 2.6679 | 999 | 0.015 | * |
| Residuals | 54 | 0.289995 | 0.0053703 |  |  |  |  |
|  |  |  |  |  |  |  |  |
| Signif. codes: 0 ‘***’ 0.001 ‘**’ 0.01 ‘*’ 0.05 ‘.’ 0.1 ‘ ’ 1 | | | |  |  |  |  |

| **Pairwise comparisons:** | |  |  |  |  |
| --- | --- | --- | --- | --- | --- |
| (Observed p-value below diagonal, permuted p-value above diagonal) | | | | | |
|  |  |  |  |  |  |
|  | Agata | Hermes | Lady Claire |  |  |
| Agata | 0.1430 | 0.212 |  |  |  |
| Hermes | 0.12797 |  | 0.881 |  |  |
| Lady Claire | 0.21947 | 0.88417 |  |  |  |

|  | T2 | T3 | T4 | T5 | T6 | T7 T | 7_Sprouts |
| --- | --- | --- | --- | --- | --- | --- | --- |
| T2 |  | 9.82E+03 | 1.06E+03 | 1.41E+03 | 2.00E+01 | 5.62E+03 | 0.210 |
| T3 | 9.85E+03 |  | 1.55E+03 | 2.56E+03 | 1.10E+02 | 6.37E+03 | 0.275 |
| T4 | 1.07E+03 | 1.57E+03 |  | 8.25E+03 | 9.80E+02 | 1.13E+03 | 0.686 |
| T5 | 1.59E+03 | 2.18E+03 | 8.03E+03 |  | 5.00E+02 | 2.13E+03 | 0.873 |
| T6 | 3.64E+01 | 1.06E+02 | 8.75E+02 | 5.28E+02 |  | 1.00E+01 | 0.045 |
| T7 | 5.61E+03 | 6.32E+03 | 1.04E+03 | 1.90E+03 | 3.16E-01 |  | 0.270 |
| T7_Sprouts | 2.1023e-01 | 2.75E+03 | 6.66E+03 | 8.54E+03 | 3.56E+02 | 2.80E+03 |  |

| **permutation MANOVA on a distance matrix** | | | | | | | |  | | | |  |  |  |  |
| --- | --- | --- | --- | --- | --- | --- | --- | --- | --- | --- | --- | --- | --- | --- | --- |
| Number of permutations: 9999 | | | |  | | | |  | | | |  |  |  |  |
|  | |  | |  | | | |  | | | |  |  |  |  |
|  | | Df | | SumsOfSqs | | | | MeanSqs | | | | F.Model | R2 | Pr(>F) |  |
| Cultivar | | 2 | | 0.3967 | | | | 0.198329 | | | | 2.1658 | 0.06949 | 4.00E-04 | *** |
| Residuals | | 58 | | 5.3111 | | | | 0.091571 | | | | 0.93051 |  |  |  |
| Total | | 60 | | 5.7078 | | | |  | | | | 1.00000 |  |  |  |
|  | |  | |  | | | |  | | | |  |  |  |  |
|  | | Df | | SumsOfSqs | | | | MeanSqs | | | | F.Model | R2 | Pr(>F) |  |
| Timepoint | | 6 | | 1.2631 | | | | 0.210518 | | | | 2.5577 | 0.2213 | 1.00E-04 | *** |
| Residuals | | 54 | | 4.4447 | | | | 0.082309 | | | | 0.7787 |  |  |  |
| Total | | 60 | | 5.7078 | | | |  | | | | 1.0000 |  |  |  |
|  | |  | |  | | | |  | | | |  |  |  |  |
| Signif. codes: 0 ‘***’ 0.001 ‘**’ 0.01 ‘*’ 0.05 ‘.’ 0.1 ‘ ’ 1 | | | | | | | |  | | | |  |  |  |  |
| **Pairwise comparisons using permutation MANOVAs on a distance matrix** | | | | | | | | | | | |  |  |  |  |
|  |  | | | |  | |  | |  | |  |  |  |  |  |
| Number of permutations: 9999 | | | | | |  | | | |  | |  |  |  |  |
|  | | |  | | |  | | | |  | |  |  |  |  |
|  | | | Agata | | | Hermes | | | |  | |  |  |  |  |
| Hermes | | | 0.0075 | | | - | | | |  | |  |  |  |  |
| Lady Claire | | | 0.0075 | | | 0.0285 | | | |  | |  |  |  |  |
|  | | |  | | |  | | | |  | |  |  |  |  |
|  | | | T2 | | | T3 | | | | T4 | | T5 | T6 | T7 |  |
| T3 | | | 0.0987 | | | - | | | | - | | - | - | - |  |
| T4 | | | 0.0007 | | | 0.0010 | | | | - | | - | - | - |  |
| T5 | | | 0.0007 | | | 0.0011 | | | | 0.0007 | | - | - | - |  |
| T6 | | | 0.0007 | | | 0.0054 | | | | 0.0010 | | 0.1521 | - | - |  |
| T7 | | | 0.0009 | | | 0.0153 | | | | 0.0010 | | 0.0280 | 0.1430 | - |  |
| T7_Sprouts | | | 0.0007 | | | 0.0016 | | | | 0.0007 | | 0.0047 | 0.2687 | 0.1307 |  |
|  | | |  | | |  | | | |  | |  |  |  |  |
| P value adjustment method: fdr | | | | | |  | | | |  | |  |  |  |  |

| **permutation CAP test on Bray Curtis dissimilarity matrix** | | | | | | |  | |  | |  |
| --- | --- | --- | --- | --- | --- | --- | --- | --- | --- | --- | --- |
| Number of permutations: 999 | | | |  | | |  | |  | |  |
|  | |  | |  | | |  | |  | |  |
|  | | Df | | SumOfSqs | | | F | | Pr(>F) | |  |
| Cultivar | | 2 | | 0.3967 | | | 2.5490 | | 0.002 | | ** |
| Timepoint | | 6 | | 1.2653 | | | 2.7103 | | 0.001 | | *** |
| Residual | | 52 | | 4.0459 | | |  | |  | |  |
|  | |  | |  | | |  | |  | |  |
| Signif. codes: 0 ‘***’ 0.001 ‘**’ 0.01 ‘*’ 0.05 ‘.’ 0.1 ‘ ’ 1 | | | | | | |  | |  | |  |
|  | |  | |  | | |  | |  | |  |
| **multivariate GLM** | | |  | |  |  | |  | |  |  |
|  |  | |  | |  |  | |  | |  |  |
|  | Res.Df | | Df.diff | | Dev | Pr(>Dev) | |  | |  |  |
| (Intercept) | 60 | |  | |  |  | |  | |  |  |
| **Timepoint** | 54 | | 6 | | 13761 | 0.01 | | ** | |  |  |
|  |  | |  | |  |  | |  | |  |  |
|  | Res.Df | | Df.diff | | Dev | Pr(>Dev) | |  | |  |  |
| (Intercept) | 60 | |  | |  |  | |  | |  |  |
| **Cultivar** | 58 | | 2 | | 3905 | 0.01 | | ** | |  |  |
|  |  | |  | |  |  | |  | |  |  |
| Signif. codes: 0 ‘***’ 0.001 ‘**’ 0.01 ‘*’ 0.05 ‘.’ 0.1 ‘ ’ 1 | | | | | |  | |  | |  |  |

**Supplementary Table S5: Taxonomic classification, relative abundance and relative abundance in % of the bacterial community of potato tubers sampled during storage time T2-7.** Specifically of the cultivars Agata, Hermes and Lady Claire. (The top ten taxa visualized in Fig**.** 4)

| **Sample** | **Genus** | **relative Abundance** | **relative Abundance [%]** |
| --- | --- | --- | --- |
| **T2** | Nocardioides | 0.11 | 10.87 |
|  | Iamia | 0.10 | 10.28 |
|  | Propionibacterium | 0.02 | 2.48 |
|  | Staphylococcus | 0.00 | 0.00 |
|  | Acinetobacter | 0.03 | 2.53 |
|  | Delftia | 0.00 | 0.00 |
|  | Escherichia-Shigella | 0.00 | 0.00 |
|  | Bacillus | 0.03 | 2.78 |
|  | Lysinibacillus | 0.00 | 0.00 |
|  | Acidothermus | 0.03 | 2.56 |
| **T3** | Nocardioides | 0.15 | 14.68 |
|  | Iamia | 0.11 | 10.76 |
|  | Propionibacterium | 0.05 | 4.86 |
|  | Staphylococcus | 0.05 | 4.86 |
|  | Acinetobacter | 0.02 | 2.50 |
|  | Delftia | 0.03 | 2.65 |
|  | Escherichia-Shigella | 0.01 | 0.98 |
|  | Bacillus | 0.00 | 0.00 |
|  | Lysinibacillus | 0.02 | 2.14 |
|  | Acidothermus | 0.03 | 2.67 |
| **T4** | Nocardioides | 0.09 | 9.27 |
|  | Iamia | 0.11 | 11.15 |
|  | Propionibacterium | 0.08 | 8.41 |
|  | Staphylococcus | 0.07 | 6.58 |
|  | Acinetobacter | 0.03 | 3.21 |
|  | Delftia | 0.02 | 2.39 |
|  | Escherichia-Shigella | 0.00 | 0.00 |
|  | Bacillus | 0.00 | 0.00 |
|  | Lysinibacillus | 0.00 | 0.00 |
|  | Acidothermus | 0.00 | 0.00 |
| **T5** | Nocardioides | 0.10 | 9.67 |
|  | Iamia | 0.09 | 9.47 |
|  | Propionibacterium | 0.13 | 13.38 |
|  | Staphylococcus | 0.08 | 7.71 |
|  | Acinetobacter | 0.04 | 3.53 |
|  | Delftia | 0.00 | 0.00 |
|  | Escherichia-Shigella | 0.00 | 0.00 |
|  | Bacillus | 0.02 | 1.90 |
|  | Lysinibacillus | 0.00 | 0.00 |
|  | Acidothermus | 0.04 | 4.31 |
| **T6** | Nocardioides | 0.11 | 10.84 |
|  | Iamia | 0.06 | 6.27 |
|  | Propionibacterium | 0.08 | 8.27 |
|  | Staphylococcus | 0.11 | 11.22 |
|  | Acinetobacter | 0.09 | 9.02 |
|  | Delftia | 0.08 | 7.63 |
|  | Escherichia-Shigella | 0.01 | 1.09 |
|  | Bacillus | 0.05 | 4.85 |
|  | Lysinibacillus | 0.07 | 6.56 |
|  | Acidothermus | 0.00 | 0.00 |
| **T7** | Nocardioides | 0.09 | 8.97 |
|  | Iamia | 0.08 | 8.28 |
|  | Propionibacterium | 0.08 | 7.75 |
|  | Staphylococcus | 0.07 | 6.93 |
|  | Acinetobacter | 0.10 | 9.69 |
|  | Delftia | 0.07 | 6.93 |
|  | Escherichia-Shigella | 0.03 | 2.77 |
|  | Bacillus | 0.05 | 5.49 |
|  | Lysinibacillus | 0.05 | 4.64 |
|  | Acidothermus | 0.03 | 2.81 |
| **T7_Sprouts** | Nocardioides | 0.06 | 6.16 |
|  | Iamia | 0.05 | 4.74 |
|  | Propionibacterium | 0.08 | 7.68 |
|  | Staphylococcus | 0.10 | 9.52 |
|  | Acinetobacter | 0.08 | 7.55 |
|  | Delftia | 0.10 | 10.13 |
|  | Escherichia-Shigella | 0.15 | 14.73 |
|  | Bacillus | 0.07 | 7.12 |
|  | Lysinibacillus | 0.05 | 4.69 |
|  | Acidothermus | 0.02 | 2.31 |

**Supplementary Table S6:** **Taxonomic classification of differentially abundant rOTUs.** Differentially abundant rOTUs of the bacterial communities based on the sequencing data from tuber of the variety Agata, Hermes and Lady Claire sampled at all sampling timepoints T2-7.

| **Differentially abundant rOTU** | **Kingdom** | **Phylum** | **Class** | **Order** | **Family** | **Genus** | **Time point** |
| --- | --- | --- | --- | --- | --- | --- | --- |
| OTU_266 | Bacteria | Bacteroidetes | Sphingobacteriia | Sphingobacteriales | Chitinophagaceae | NA | T2 |
| OTU_2713 | Bacteria | Proteobacteria | Deltaproteobacteria | Oligoflexales | Oligoflexaceae | NA | T2 |
| OTU_386 | Bacteria | Bacteroidetes | Sphingobacteriia | Sphingobacteriales | Chitinophagaceae | NA | T2 |
| OTU_7289 | Bacteria | Acidobacteria | Solibacteres | Solibacterales | Solibacteraceae (Subgroup3) | NA | T2 |
| OTU_78 | Bacteria | Bacteroidetes | Cytophagia | Cytophagales | Cytophagaceae | NA | T2 |
| OTU_873 | Bacteria | Proteobacteria | Alphaproteobacteria | Rhizobiales | Hyphomicrobiaceae | NA | T2 |
| OTU_9397 | Bacteria | Chloroflexi | TK10 | NA | NA | NA | T2 |
| OTU_1506 | Bacteria | Bacteroidetes | Sphingobacteriia | Sphingobacteriales | Chitinophagaceae | NA | T3 |
| OTU_30 | Bacteria | Saccharibacteria | NA | NA | NA | NA | T3 |
| OTU_33 | Bacteria | Proteobacteria | Betaproteobacteria | Burkholderiales | Comamonadaceae | NA | T3 |
| OTU_109 | Bacteria | Actinobacteria | Actinobacteria | Propionibacteriales | Propionibacteriaceae | Propionibacterium | T4 |
| OTU_163 | Bacteria | Proteobacteria | Gammaproteobacteria | Enterobacteriales | Enterobacteriaceae | NA | T4 |
| OTU_319 | Bacteria | Actinobacteria | Acidimicrobiia | Acidimicrobiales | Iamiaceae | Iamia | T4 |
| OTU_2813 | Bacteria | Actinobacteria | Actinobacteria | Frankiales | Acidothermaceae | Acidothermus | T5 |
| OTU_69 | Bacteria | Actinobacteria | Actinobacteria | Propionibacteriales | Nocardioidaceae | Nocardioides | T6 |
| OTU_103 | Bacteria | Proteobacteria | Gammaproteobacteria | Enterobacteriales | Enterobacteriaceae | Escherichia-Shigella | T7 |
| OTU_13 | Bacteria | Proteobacteria | Gammaproteobacteria | Pseudomonadales | Moraxellaceae | Acinetobacter | T7 |
| OTU_4 | Bacteria | Firmicutes | Bacilli | Bacillales | Staphylococcaceae | Staphylococcus | T7 |
| OTU_48 | Bacteria | Firmicutes | Bacilli | Bacillales | Planococcaceae | Lysinibacillus | T7 |
| OTU_544 | Bacteria | Proteobacteria | Gammaproteobacteria | Enterobacteriales | Enterobacteriaceae | Escherichia-Shigella | T7 |
| OTU_6 | Bacteria | Proteobacteria | Betaproteobacteria | Burkholderiales | Comamonadaceae | Delftia | T7 |
| OTU_679 | Bacteria | Firmicutes | Bacilli | Bacillales | Bacillaceae | Bacillus | T7 |

**Supplementary Table S7: Taxonomic classification of differentially abundant rOTUs**. Differentially abundant rOTUs of the bacterial communities based on the sequencing data from tuber of the variety Agata, Hermes, Fabiola and Lady Claire sampled at sampling timepoints T2, T6 and T7 associated with the storage stabilities short, medium and long.

| **Differentially abundant rOTU** | **Kingdom** | **Phylum** | **Class** | **Order** | **Family** | **Genus** | **Storage stability** | **Time point** |
| --- | --- | --- | --- | --- | --- | --- | --- | --- |
| OTU_102 | Bacteria | Proteobacteria | Gammaproteobacteria | Xanthomonadales | Xanthomonadaceae | NA | long | T2 |
| OTU_133 | Bacteria | Bacteroidetes | Sphingobacteriia | Sphingobacteriales | Chitinophagaceae | Terrimonas | long | T2 |
| OTU_1659 | Bacteria | Proteobacteria | Deltaproteobacteria | Bdellovibrionales | Bacteriovoracaceae | Peredibacter | long | T2 |
| OTU_188 | Bacteria | Proteobacteria | Gammaproteobacteria | Xanthomonadales | XanthomonadalesIncertaeSedis | NA | long | T2 |
| OTU_1973 | Bacteria | Proteobacteria | Gammaproteobacteria | Legionellales | Legionellaceae | Legionella | medium | T2 |
| OTU_353 | Bacteria | Bacteroidetes | Cytophagia | Cytophagales | Cytophagaceae | Ohtaekwangia | medium | T2 |
| OTU_380 | Bacteria | Bacteroidetes | Cytophagia | Cytophagales | Cytophagaceae | NA | medium | T2 |
| OTU_4509 | Bacteria | Firmicutes | Bacilli | Bacillales | Paenibacillaceae | Paenibacillus | medium | T2 |
| OTU_68182 | Bacteria | Proteobacteria | Alphaproteobacteria | Rhodobacterales | Rhodobacteraceae | NA | medium | T2 |
| OTU_104 | Bacteria | Proteobacteria | Gammaproteobacteria | Cellvibrionales | Cellvibrionaceae | Cellvibrio | short | T2 |
| OTU_1255 | Bacteria | Acidobacteria | Holophagae | Subgroup10 | ABS-19 | NA | short | T2 |
| OTU_1291 | Bacteria | Bacteroidetes | Cytophagia | Cytophagales | Cytophagaceae | Ohtaekwangia | short | T2 |
| OTU_140 | Bacteria | Proteobacteria | Alphaproteobacteria | Caulobacterales | Caulobacteraceae | Brevundimonas | short | T2 |
| OTU_1406 | Bacteria | Chloroflexi | Chloroflexia | Herpetosiphonales | Herpetosiphonaceae | Herpetosiphon | short | T2 |
| OTU_2 | Bacteria | Proteobacteria | Betaproteobacteria | Burkholderiales | Burkholderiaceae | Burkholderia-Paraburkholderia | short | T2 |
| OTU_47 | Bacteria | Bacteroidetes | Sphingobacteriia | Sphingobacteriales | Sphingobacteriaceae | Pedobacter | short | T2 |
| OTU_783 | Bacteria | Firmicutes | Bacilli | Bacillales | Alicyclobacillaceae | Tumebacillus | short | T2 |
| OTU_829 | Bacteria | Acidobacteria | Holophagae | Subgroup7 | NA | NA | short | T2 |
| OTU_1321 | Bacteria | Bacteroidetes | Sphingobacteriia | Sphingobacteriales | NS11-12marinegroup | NA | long | T6 |
| OTU_14 | Bacteria | Bacteroidetes | Flavobacteriia | Flavobacteriales | Flavobacteriaceae | Flavobacterium | long | T6 |
| OTU_1562 | Bacteria | Actinobacteria | Thermoleophilia | Gaiellales | NA | NA | long | T6 |
| OTU_250 | Bacteria | Proteobacteria | Betaproteobacteria | Burkholderiales | Oxalobacteraceae | Massilia | long | T6 |
| OTU_2512 | Bacteria | Proteobacteria | Betaproteobacteria | Burkholderiales | Oxalobacteraceae | NA | long | T6 |
| OTU_293 | Bacteria | Bacteroidetes | Cytophagia | Cytophagales | Cytophagaceae | NA | long | T6 |
| OTU_308 | Bacteria | Bacteroidetes | Cytophagia | Cytophagales | Cytophagaceae | NA | long | T6 |
| OTU_380 | Bacteria | Bacteroidetes | Cytophagia | Cytophagales | Cytophagaceae | NA | long | T6 |
| OTU_47 | Bacteria | Bacteroidetes | Sphingobacteriia | Sphingobacteriales | Sphingobacteriaceae | Pedobacter | long | T6 |
| OTU_4963 | Bacteria | Proteobacteria | Alphaproteobacteria | Sphingomonadales | Sphingomonadaceae | Sphingomonas | long | T6 |
| OTU_594 | Bacteria | Actinobacteria | Thermoleophilia | Gaiellales | NA | NA | long | T6 |
| OTU_602 | Bacteria | Actinobacteria | Thermoleophilia | Gaiellales | NA | NA | long | T6 |
| OTU_7865 | Bacteria | Actinobacteria | Actinobacteria | Corynebacteriales | Mycobacteriaceae | Mycobacterium | long | T6 |
| OTU_131 | Bacteria | Actinobacteria | Actinobacteria | Streptosporangiales | Thermomonosporaceae | NA | medium | T6 |
| OTU_29205 | Bacteria | Firmicutes | Bacilli | Bacillales | Paenibacillaceae | Aneurinibacillus | medium | T6 |
| OTU_343 | Bacteria | Proteobacteria | Deltaproteobacteria | Myxococcales | NA | NA | medium | T6 |
| OTU_5027 | Bacteria | Proteobacteria | Alphaproteobacteria | Rhodospirillales | Rhodospirillaceae | Dongia | medium | T6 |
| OTU_505 | Bacteria | Proteobacteria | Alphaproteobacteria | Rhodospirillales | RhodospirillalesIncertaeSedis | Reyranella | medium | T6 |
| OTU_588 | Bacteria | Proteobacteria | Alphaproteobacteria | Rhizobiales | Xanthobacteraceae | Variibacter | medium | T6 |
| OTU_77 | Bacteria | Actinobacteria | Actinobacteria | Micrococcales | Microbacteriaceae | Rudaibacter | medium | T6 |
| OTU_899 | Bacteria | Chloroflexi | TK10 | NA | NA | NA | medium | T6 |
| OTU_1009 | Bacteria | Proteobacteria | Gammaproteobacteria | Xanthomonadales | Xanthomonadaceae | NA | short | T6 |
| OTU_15 | Bacteria | Proteobacteria | Gammaproteobacteria | Pseudomonadales | Pseudomonadaceae | Pseudomonas | short | T6 |
| OTU_194 | Bacteria | Proteobacteria | Alphaproteobacteria | Sphingomonadales | Sphingomonadaceae | Novosphingobium | short | T6 |
| OTU_38 | Bacteria | Proteobacteria | Betaproteobacteria | Burkholderiales | Oxalobacteraceae | Massilia | short | T6 |
| OTU_51 | Bacteria | Proteobacteria | Alphaproteobacteria | Sphingomonadales | Sphingomonadaceae | Sphingomonas | short | T6 |
| OTU_69 | Bacteria | Actinobacteria | Actinobacteria | Propionibacteriales | Nocardioidaceae | Nocardioides | short | T6 |
| OTU_883 | Bacteria | Proteobacteria | Alphaproteobacteria | Sphingomonadales | Ellin6055 | NA | short | T6 |
| OTU_957 | Bacteria | Bacteroidetes | Sphingobacteriia | Sphingobacteriales | Chitinophagaceae | Parasegetibacter | short | T6 |
| OTU_1160 | Bacteria | Proteobacteria | Alphaproteobacteria | Caulobacterales | Caulobacteraceae | Asticcacaulis | long | T7 |
| OTU_132 | Bacteria | Proteobacteria | Alphaproteobacteria | Sphingomonadales | NA | NA | long | T7 |
| OTU_1371 | Bacteria | Saccharibacteria | NA | NA | NA | NA | long | T7 |
| OTU_153 | Bacteria | Proteobacteria | Betaproteobacteria | Methylophilales | Methylophilaceae | NA | long | T7 |
| OTU_359 | Bacteria | Actinobacteria | Thermoleophilia | Solirubrobacterales | Patulibacteraceae | Patulibacter | long | T7 |
| OTU_476 | Bacteria | Actinobacteria | Actinobacteria | Micrococcales | Promicromonosporaceae | Promicromonospora | long | T7 |
| OTU_104 | Bacteria | Proteobacteria | Gammaproteobacteria | Cellvibrionales | Cellvibrionaceae | Cellvibrio | medium | T7 |
| OTU_131 | Bacteria | Actinobacteria | Actinobacteria | Streptosporangiales | Thermomonosporaceae | NA | medium | T7 |
| OTU_2004 | Bacteria | Chloroflexi | TK10 | NA | NA | NA | medium | T7 |
| OTU_216 | Bacteria | Bacteroidetes | Flavobacteriia | Flavobacteriales | Flavobacteriaceae | Flavobacterium | medium | T7 |
| OTU_340 | Bacteria | Proteobacteria | Gammaproteobacteria | Pseudomonadales | Moraxellaceae | NA | medium | T7 |
| OTU_47 | Bacteria | Bacteroidetes | Sphingobacteriia | Sphingobacteriales | Sphingobacteriaceae | Pedobacter | medium | T7 |
| OTU_512 | Bacteria | Proteobacteria | Betaproteobacteria | Burkholderiales | Comamonadaceae | Aquabacterium | medium | T7 |
| OTU_513 | Bacteria | Chloroflexi | Chloroflexia | Chloroflexales | Roseiflexaceae | Roseiflexus | medium | T7 |
| OTU_584 | Bacteria | Chloroflexi | Chloroflexia | Chloroflexales | Roseiflexaceae | Roseiflexus | medium | T7 |
| OTU_641 | Bacteria | Proteobacteria | Alphaproteobacteria | Rhizobiales | Phyllobacteriaceae | NA | medium | T7 |
| OTU_89 | Bacteria | Bacteroidetes | Flavobacteriia | Flavobacteriales | Flavobacteriaceae | Flavobacterium | medium | T7 |
| OTU_10 | Bacteria | Proteobacteria | Betaproteobacteria | Burkholderiales | Burkholderiaceae | Ralstonia | short | T7 |
| OTU_103 | Bacteria | Proteobacteria | Gammaproteobacteria | Enterobacteriales | Enterobacteriaceae | Escherichia-Shigella | short | T7 |
| OTU_13 | Bacteria | Proteobacteria | Gammaproteobacteria | Pseudomonadales | Moraxellaceae | Acinetobacter | short | T7 |
| OTU_134 | Bacteria | Actinobacteria | Actinobacteria | Propionibacteriales | Nocardioidaceae | Nocardioides | short | T7 |
| OTU_1458 | Bacteria | Proteobacteria | Deltaproteobacteria | Myxococcales | Phaselicystidaceae | Phaselicystis | short | T7 |
| OTU_19 | Bacteria | Proteobacteria | Alphaproteobacteria | Rhizobiales | Phyllobacteriaceae | Mesorhizobium | short | T7 |
| OTU_256 | Bacteria | Firmicutes | Bacilli | Bacillales | Planococcaceae | Solibacillus | short | T7 |
| OTU_262 | Bacteria | Firmicutes | Bacilli | NA | NA | NA | short | T7 |
| OTU_4 | Bacteria | Firmicutes | Bacilli | Bacillales | Staphylococcaceae | Staphylococcus | short | T7 |
| OTU_679 | Bacteria | Firmicutes | Bacilli | Bacillales | Bacillaceae | Bacillus | short | T7 |
| OTU_73 | Bacteria | Proteobacteria | Betaproteobacteria | Burkholderiales | Burkholderiaceae | Burkholderia-Paraburkholderia | short | T7 |
| OTU_832 | Bacteria | Actinobacteria | MB-A2-108 | NA | NA | NA | short | T7 |

**Supplementary Table S8: Taxonomic classification of rOTUs correlated with spearman product moment.** Correlated rOTUs of the bacterial communities based on the sequencing data from tuber of the variety Agata, Hermes, Fabiola and Lady Claire sampled at sampling timepoints T2, T6 and T7.

| **OTU** | **Kingdom** | **Phylum** | **Class** | **Order** | **Family** | **Genus** | **resr** | **Time point** |
| --- | --- | --- | --- | --- | --- | --- | --- | --- |
| OTU_297 | Bacteria | Proteobacteria | Betaproteobacteria | Burkholderiales | Oxalobacteraceae | NA | -0.3524 | T2 |
| OTU_2846 | Bacteria | Actinobacteria | Actinobacteria | Micrococcales | Microbacteriaceae | Microbacterium | -0.3719 | T2 |
| OTU_118 | Bacteria | Actinobacteria | Actinobacteria | Micromonosporales | Micromonosporaceae | NA | -0.3577 | T2 |
| OTU_380 | Bacteria | Bacteroidetes | Cytophagia | Cytophagales | Cytophagaceae | NA | 0.4163 | T2 |
| OTU_2135 | Bacteria | Actinobacteria | Actinobacteria | Pseudonocardiales | Pseudonocardiaceae | NA | -0.3655 | T2 |
| OTU_1320 | Bacteria | Actinobacteria | Actinobacteria | Micromonosporales | Micromonosporaceae | Actinoplanes | -0.4574 | T2 |
| OTU_537 | Bacteria | Bacteroidetes | Cytophagia | Cytophagales | Cytophagaceae | NA | -0.3584 | T2 |
| OTU_1174 | Bacteria | Actinobacteria | Thermoleophilia | Gaiellales | NA | NA | -0.3565 | T2 |
| OTU_1406 | Bacteria | Chloroflexi | Chloroflexia | Herpetosiphonales | Herpetosiphonaceae | Herpetosiphon | -0.4539 | T2 |
| OTU_275 | Bacteria | Bacteroidetes | Flavobacteriia | Flavobacteriales | Flavobacteriaceae | Flavobacterium | 0.3647 | T6 |
| OTU_47 | Bacteria | Bacteroidetes | Sphingobacteriia | Sphingobacteriales | Sphingobacteriaceae | Pedobacter | 0.4955 | T6 |
| OTU_505 | Bacteria | Proteobacteria | Alphaproteobacteria | Rhodospirillales | RhodospirillalesIncertaeSedis | Reyranella | 0.3770 | T6 |
| OTU_588 | Bacteria | Proteobacteria | Alphaproteobacteria | Rhizobiales | Xanthobacteraceae | Variibacter | 0.3586 | T6 |
| OTU_85 | Bacteria | Proteobacteria | Alphaproteobacteria | Sphingomonadales | Sphingomonadaceae | Sphingopyxis | 0.4930 | T6 |
| OTU_121 | Bacteria | Bacteroidetes | Sphingobacteriia | Sphingobacteriales | Chitinophagaceae | Parasegetibacter | 0.3504 | T6 |
| OTU_602 | Bacteria | Actinobacteria | Thermoleophilia | Gaiellales | NA | NA | 0.3898 | T6 |
| OTU_957 | Bacteria | Bacteroidetes | Sphingobacteriia | Sphingobacteriales | Chitinophagaceae | Parasegetibacter | -0.4339 | T6 |
| OTU_747 | Bacteria | Bacteroidetes | Sphingobacteriia | Sphingobacteriales | Chitinophagaceae | Ferruginibacter | -0.3711 | T6 |
| OTU_7865 | Bacteria | Actinobacteria | Actinobacteria | Corynebacteriales | Mycobacteriaceae | Mycobacterium | 0.3854 | T6 |
| OTU_380 | Bacteria | Bacteroidetes | Cytophagia | Cytophagales | Cytophagaceae | NA | 0.4120 | T6 |
| OTU_1562 | Bacteria | Actinobacteria | Thermoleophilia | Gaiellales | NA | NA | 0.3860 | T6 |
| OTU_14 | Bacteria | Bacteroidetes | Flavobacteriia | Flavobacteriales | Flavobacteriaceae | Flavobacterium | 0.4174 | T6 |
| OTU_5027 | Bacteria | Proteobacteria | Alphaproteobacteria | Rhodospirillales | Rhodospirillaceae | Dongia | 0.4473 | T6 |
| OTU_4 | Bacteria | Firmicutes | Bacilli | Bacillales | Staphylococcaceae | Staphylococcus | -0.4784 | T7 |
| OTU_152 | Bacteria | Proteobacteria | Gammaproteobacteria | Xanthomonadales | Xanthomonadaceae | NA | -0.3602 | T7 |
| OTU_43 | Bacteria | Bacteroidetes | Cytophagia | Cytophagales | Cytophagaceae | NA | -0.3766 | T7 |
| OTU_13 | Bacteria | Proteobacteria | Gammaproteobacteria | Pseudomonadales | Moraxellaceae | Acinetobacter | -0.4371 | T7 |
| OTU_73 | Bacteria | Proteobacteria | Betaproteobacteria | Burkholderiales | Burkholderiaceae | Burkholderia-Paraburkholderia | -0.3675 | T7 |
| OTU_288 | Bacteria | Firmicutes | Bacilli | Bacillales | Thermoactinomycetaceae | Thermoflavimicrobium | -0.3947 | T7 |
| OTU_19 | Bacteria | Proteobacteria | Alphaproteobacteria | Rhizobiales | Phyllobacteriaceae | Mesorhizobium | -0.5621 | T7 |
| OTU_216 | Bacteria | Bacteroidetes | Flavobacteriia | Flavobacteriales | Flavobacteriaceae | Flavobacterium | 0.3941 | T7 |
| OTU_235 | Bacteria | Proteobacteria | Gammaproteobacteria | Xanthomonadales | Xanthomonadaceae | Pseudoxanthomonas | -0.3510 | T7 |
| OTU_105 | Bacteria | Proteobacteria | Gammaproteobacteria | Xanthomonadales | Xanthomonadaceae | Dyella | -0.4735 | T7 |
| OTU_103 | Bacteria | Proteobacteria | Gammaproteobacteria | Enterobacteriales | Enterobacteriaceae | Escherichia-Shigella | -0.5704 | T7 |
| OTU_813 | Bacteria | Gemmatimonadetes | Gemmatimonadetes | Gemmatimonadales | Gemmatimonadaceae | Gemmatimonas | -0.3570 | T7 |
| OTU_1160 | Bacteria | Proteobacteria | Alphaproteobacteria | Caulobacterales | Caulobacteraceae | Asticcacaulis | 0.4237 | T7 |
| OTU_688 | Bacteria | Bacteroidetes | Cytophagia | Cytophagales | Cytophagaceae | NA | 0.3629 | T7 |
| OTU_162 | Bacteria | Actinobacteria | Actinobacteria | Glycomycetales | Glycomycetaceae | Glycomyces | -0.4361 | T7 |
| OTU_252 | Bacteria | Proteobacteria | Alphaproteobacteria | Rhizobiales | Brucellaceae | Ochrobactrum | -0.4054 | T7 |
| OTU_679 | Bacteria | Firmicutes | Bacilli | Bacillales | Bacillaceae | Bacillus | -0.5495 | T7 |
| OTU_1458 | Bacteria | Proteobacteria | Deltaproteobacteria | Myxococcales | Phaselicystidaceae | Phaselicystis | -0.3696 | T7 |
| OTU_262 | Bacteria | Firmicutes | Bacilli | NA | NA | NA | -0.3846 | T7 |
| OTU_349 | Bacteria | Proteobacteria | Betaproteobacteria | Burkholderiales | Comamonadaceae | NA | 0.3859 | T7 |
| OTU_1240 | Bacteria | Proteobacteria | Alphaproteobacteria | Sphingomonadales | NA | NA | 0.3609 | T7 |
| OTU_153 | Bacteria | Proteobacteria | Betaproteobacteria | Methylophilales | Methylophilaceae | NA | 0.4142 | T7 |
| OTU_359 | Bacteria | Actinobacteria | Thermoleophilia | Solirubrobacterales | Patulibacteraceae | Patulibacter | 0.3820 | T7 |
| OTU_256 | Bacteria | Firmicutes | Bacilli | Bacillales | Planococcaceae | Solibacillus | -0.4585 | T7 |
| OTU_861 | Bacteria | Firmicutes | Bacilli | Lactobacillales | Streptococcaceae | Streptococcus | -0.4620 | T7 |
| OTU_261 | Bacteria | Proteobacteria | Betaproteobacteria | Nitrosomonadales | Nitrosomonadaceae | NA | -0.3605 | T7 |
| OTU_1024 | Bacteria | Proteobacteria | Betaproteobacteria | TRA3-20 | NA | NA | -0.4624 | T7 |

**Supplementary Table S9: Taxonomic classification of key OTUs.** Key OTUs calculated with the random forest function and with correlation matrix based on spearman product moment.

| **key OTU** | **Kingdom** | **Phylum** | **Class** | **Order** | **Family** | **Genus** | **Storage stability** | **Time point** |
| --- | --- | --- | --- | --- | --- | --- | --- | --- |
| OTU_1406 | Bacteria | Chloroflexi | Chloroflexia | Herpetosiphonales | Herpetosiphonaceae | Herpetosiphon | short | T2 |
| OTU_957 | Bacteria | Bacteroidetes | Sphingobacteriia | Sphingobacteriales | Chitinophagaceae | Parasegetibacter | short | T6 |
| OTU_103 | Bacteria | Proteobacteria | Gammaproteobacteria | Enterobacteriales | Enterobacteriaceae | Escherichia-Shigella | short | T7 |
| OTU_13 | Bacteria | Proteobacteria | Gammaproteobacteria | Pseudomonadales | Moraxellaceae | Acinetobacter | short | T7 |
| OTU_1458 | Bacteria | Proteobacteria | Deltaproteobacteria | Myxococcales | Phaselicystidaceae | Phaselicystis | short | T7 |
| OTU_19 | Bacteria | Proteobacteria | Alphaproteobacteria | Rhizobiales | Phyllobacteriaceae | Mesorhizobium | short | T7 |
| OTU_256 | Bacteria | Firmicutes | Bacilli | Bacillales | Planococcaceae | Solibacillus | short | T7 |
| OTU_4 | Bacteria | Firmicutes | Bacilli | Bacillales | Staphylococcaceae | Staphylococcus | short | T7 |
| OTU_679 | Bacteria | Firmicutes | Bacilli | Bacillales | Bacillaceae | Bacillus | short | T7 |
| OTU_73 | Bacteria | Proteobacteria | Betaproteobacteria | Burkholderiales | Burkholderiaceae | Burkholderia-Paraburkholderia | short | T7 |
| OTU_380 | Bacteria | Bacteroidetes | Cytophagia | Cytophagales | Cytophagaceae | NA | medium | T2 |
| OTU_5027 | Bacteria | Proteobacteria | Alphaproteobacteria | Rhodospirillales | Rhodospirillaceae | Dongia | medium | T6 |
| OTU_505 | Bacteria | Proteobacteria | Alphaproteobacteria | Rhodospirillales | RhodospirillalesIncertae Sedis | Reyranella | medium | T6 |
| OTU_588 | Bacteria | Proteobacteria | Alphaproteobacteria | Rhizobiales | Xanthobacteraceae | Variibacter | medium | T6 |
| OTU_216 | Bacteria | Bacteroidetes | Flavobacteriia | Flavobacteriales | Flavobacteriaceae | Flavobacterium | medium | T7 |
| OTU_14 | Bacteria | Bacteroidetes | Flavobacteriia | Flavobacteriales | Flavobacteriaceae | Flavobacterium | long | T6 |
| OTU_1562 | Bacteria | Actinobacteria | Thermoleophilia | Gaiellales | NA | NA | long | T6 |
| OTU_380 | Bacteria | Bacteroidetes | Cytophagia | Cytophagales | Cytophagaceae | NA | long | T6 |
| OTU_47 | Bacteria | Bacteroidetes | Sphingobacteriia | Sphingobacteriales | Sphingobacteriaceae | Pedobacter | long | T6 |
| OTU_602 | Bacteria | Actinobacteria | Thermoleophilia | Gaiellales | NA | NA | long | T6 |
| OTU_7865 | Bacteria | Actinobacteria | Actinobacteria | Corynebacteriales | Mycobacteriaceae | Mycobacterium | long | T6 |
| OTU_1160 | Bacteria | Proteobacteria | Alphaproteobacteria | Caulobacterales | Caulobacteraceae | Asticcacaulis | long | T7 |
| OTU_153 | Bacteria | Proteobacteria | Betaproteobacteria | Methylophilales | Methylophilaceae | NA | long | T7 |
| OTU_359 | Bacteria | Actinobacteria | Thermoleophilia | Solirubrobacterales | Patulibacteraceae | Patulibacter | long | T7 |

**Supplementary Table S10:** **Investigation of the potato tuber sprouting behavior after treatment with 218 different bacterial cultures in an *in-vitro* potato tuber sprouting assay.** Tuber discs of the potato variety Lady Claire were used. When tuber buds treated with maleic hydrazine reached stage 01, the assay was closed and the sum of all growth stages of each replicate was calculated. Afterwards the mean of the sum of three replicates was visualized with a color gradient. The color gradient shows which treatments have led to a sprouting promotion or inhibition in comparison to the assay control. The assay control gibberellic acid represents an effective sprouting promotion whereas maleic hydrazine is used as the negative control. 10% sterile tryptic soy broth (used as the medium for isolate cultures) and sterile water was used as a neutral control.

| **Treatment** | **AIT ID** | **Repetition 1** | **Repetition 2** |
| --- | --- | --- | --- |
| Gibberellic acid |  | 10.67 | 5.00 |
| Maleic haydrazine |  | 0.67 | 0.67 |
| H₂O |  | 3.33 | 3.00 |
| 10% TSB |  | 1.67 | 4.00 |
| *Curtobacterium* sp. | 1109 | 3.33 | 1.33 |
| *Micrococcus* sp. | 1110 | 5.00 | 0.67 |
| *Bacillus* sp. | 1111 | 3.33 | 1.00 |
| *Staphylococcus* sp. | 1112 | 5.33 | 1.00 |
| *Bacillus* sp. | 1113 | 1.33 | 0.67 |
| *Bacillus* sp. | 1114 | 2.00 | 1.67 |
| *Bacillus* sp. | 1115 | 7.00 | 1.67 |
| *Bacillus* sp. | 1116 | 3.33 | 0.67 |
| *Bacillus* sp. | 1117 | 0.00 | 1.67 |
| *Staphylococcus* sp. | 1118 | 0.67 | 0.67 |
| *Arthrobacter* sp. | 1119 | 1.67 | 0.67 |
| *Arthrobacter* sp. | 1120 | 2.33 | 1.67 |
| *Rhodococcus* sp. | 1121 | 4.67 | 2.00 |
| *Bacillus* sp. | 1122 | 7.00 | 2.00 |
| *Microbacterium* sp. | 1123 | 7.00 | 0.33 |

| **Treatment** | **AIT ID** | **Repetition 1** | **Repetition 2** |
| --- | --- | --- | --- |
| Gibberellic Acid |  | 4.33 | 5.33 |
| Maleic Acid |  | 0.33 | 0.33 |
| H₂O |  | 0.00 | 0.00 |
| 10% TSB |  | 1.00 | 2.00 |
| *Bacillus* sp. | 1124 | 0.33 | 1.33 |
| *Microbacterium* sp. | 1125 | 0.00 | 0.00 |
| *Bacillus* sp. | 1126 | 0.00 | 0.00 |
| *Staphylococcus* sp. | 1127 | 1.33 | 0.00 |
| *Microbacterium* sp. | 1128 | 2.00 | 0.00 |
| *Bacillus* sp. | 1129 | 1.00 | 0.00 |
| Curtobacterium sp. | 1130 | 2.33 | 1.00 |
| *Bacillus* sp. | 1131 | 0.00 | 1.33 |
| *Arthrobacter* sp. | 1132 | 1.00 | 0.33 |
| *Bacillus* sp. | 1134 | 2.33 | 2.00 |
| *Arthrobacter* sp. | 1136 | 0.00 | 0.00 |
| *Bacillus* sp. | 1137 | 1.33 | 0.33 |
| *Bacillus* sp. | 1138 | 0.00 | 0.00 |
| *Pantoea* sp. | 1139 | 0.00 | 0.33 |
| *Pantoea* sp. | 1140 | 0.67 | 0.33 |
| *Pseudomonas* sp. | 1141 | 2.33 | 0.67 |
| *Arthrobacter* sp. | 1143 | 0.00 | 0.00 |
| *Stenotrophomonas* sp. | 1144 | 0.00 | 0.00 |
| *Stenotrophomonas* sp. | 1145 | 0.33 | 0.67 |
| *Bacillus* sp. | 1146 | 0.00 | 0.00 |
| *Staphylococcus* sp. | 1147 | 0.00 | 0.33 |
| *Arthrobacter* sp. | 1148 | 0.67 | 1.00 |
| *Bacillus* sp. | 1149 | 0.00 | 0.00 |
| *Pantoea* sp. | 1150 | 2.33 | 2.00 |
| *Microbacterium* sp. | 1151 | 0.00 | 0.00 |

| **Treatment** | **AIT ID** | **Repetition 1** | **Repetition 2** |
| --- | --- | --- | --- |
| Gibberellic Acid |  | 5.33 | 5.67 |
| Maleic Acid |  | 0.33 | 0.33 |
| H₂O |  | 3.33 | 3.33 |
| 10% TSB |  | 2.33 | 2.00 |
| *Chryseobacterium* sp. | 1152 | 2.00 | 0.00 |
| *Microbacterium* sp. | 1153 | 2.00 | 3.33 |
| *Nocardioides* sp. | 1154 | 1.00 | 1.00 |
| *Lysinibacillus* sp. | 1157 | 2.33 | 2.00 |
| *Bacillus* sp. | 1158 | 2.00 | 0.00 |

| **Treatment** | **AIT ID** | **Repetition 1** | **Repetition 2** |
| --- | --- | --- | --- |
| Gibberellic acid |  | 14.00 | 2.67 |
| Maleic hydrazine |  | 0.33 | 0.00 |
| H₂O |  | 4.67 | 1.00 |
| 10% TSB |  | 7.67 | 4.00 |
| *Arthrobacter* sp. | 1156 | 3.00 | 0.00 |
| *Bacillus* sp. | 1160 | 1.67 | 0.00 |
| *Arthrobacter* sp. | 1161 | 4.67 | 0.33 |
| *Pseudomonas* sp. | 1162 | 3.00 | 3.33 |
| *Microbacterium* sp. | 1163 | 0.00 | 1.00 |
| *Planococcaceae incertae sedis* | 1164 | 0.00 | 3.00 |
| *Bacillus* sp. | 1185 | 0.00 | 2.67 |
| *Staphylococcus* sp. | 1186 | 4.00 | 1.33 |
| *Bacillus* sp. | 1188 | 0.33 | 0.00 |
| *Arthrobacter* sp. | 1189 | 2.33 | 0.00 |
| *Bacillus* sp. | 1190 | 0.00 | 2.00 |
| *Kluyvera* sp. | 1191 | 0.00 | 0.00 |
| *Bacillus* sp. | 1192 | 0.00 | 0.00 |
| *Arthrobacter* sp. | 1193 | 0.00 | 1.00 |
| *Staphylococcus* sp. | 1194 | 0.67 | 0.00 |
| *Pantoea* sp. | 1195 | 0.00 | 0.00 |
| *Bacillus* sp. | 1197 | 2.00 | 2.33 |
| *Bacillus* sp. | 1198 | 2.00 | 2.33 |
| *Bacillus* sp. | 1200 | 0.33 | 1.00 |

| **Treatment** | **AIT ID** | **Repetition 1** | **Repetition 2** |
| --- | --- | --- | --- |
| Gibberellic acid |  | 6.00 | 8.67 |
| Maleic hydrazine |  | 0.33 | 0.33 |
| H₂O |  | 0.00 | 8.67 |
| 10% TSB |  | 6.67 | 12.00 |
| *Flavobacterium* sp. | 1165 | 4.00 | 3.00 |
| *Staphylococcus* sp. | 1166 | 1.67 | 3.00 |
| *Arthrobacter* sp. | 1167 | 0.00 | 0.00 |
| *Lysinibacillus* sp. | 1168 | 0.67 | 0.33 |
| *Rhodococcus* sp. | 1169 | 4.33 | 0.67 |
| Staphylococcus sp. | 1170 | 0.00 | 0.67 |
| *Arthrobacter* sp. | 1171 | 1.67 | 0.00 |
| *Rhizobium* sp. | 1172 | 1.00 | 6.00 |
| *Pseudomonas* sp. | 1173 | 0.33 | 2.33 |
| *Planococcaceae incertae sedis* | 1174 | 0.00 | 1.33 |
| *Pantoea* sp. | 1175 | 0.00 | 3.33 |
| *Pseudomonas* sp. | 1176 | 3.67 | 1.00 |
| *Staphylococcus* sp. | 1177 | 0.00 | 4.00 |
| *Bacillus* sp. | 1178 | 3.33 | 0.00 |
| *Planococcaceae incertae sedis* | 1179 | 0.00 | 1.67 |
| *Bacillus* sp. | 1180 | 0.67 | 0.00 |
| *Flavobacterium* sp. | 1181 | 0.33 | 0.00 |
| *Bacillus* sp. | 1182 | 0.00 | 0.33 |
| *Bacillus* sp. | 1184 | 0.00 | 0.00 |
| *Arthrobacter* sp. | 1159 | 1.67 | 1.00 |

| **Treatment** | **AIT ID** | **Repetition 1** | **Repetition 2** |
| --- | --- | --- | --- |
| Gibberellic acid |  | 20.00 | 11.67 |
| Maleic hydrazine |  | 0.33 | 0.67 |
| H₂O |  | 13.67 | 8.00 |
| 10% TSB |  | 5.67 | 6.67 |
| *Bacillus* sp. | 1196 | 10.00 | 11.67 |
| *Bacillus* sp. | 1199 | 6.00 | 5.67 |
| *Staphylococcus* sp. | 1226 | 4.00 | 1.67 |
| *Microbacterium* sp. | 1227 | 8.33 | 6.00 |
| *Staphylococcus* sp. | 1228 | 8.00 | 7.67 |
| *Arthrobacter* sp. | 1229 | 8.33 | 9.00 |
| *Rhodococcus* sp. | 1230 | 8.33 | 1.33 |
| *Microbacterium* sp. | 1231 | 4.67 | 4.67 |
| *Staphylococcus* sp. | 1232 | 7.00 | 6.67 |
| *Microbacterium* sp. | 1233 | 6.00 | 5.33 |
| *Agrococcus* sp. | 1234 | 11.00 | 8.33 |
| *Rhizobium* sp. | 1235 | 9.00 | 2.67 |
| *Microbacterium* sp. | 1237 | 5.33 | 3.00 |
| *Variovorax* sp. | 1238 | 4.67 | 7.33 |
| *Bacillus* sp. | 1239 | 7.00 | 7.33 |
| *Agromyces* sp. | 1240 | 11.33 | 10.67 |
| *Arthrobacter* sp. | 1241 | 7.00 | 5.00 |
| *Brevibacterium* sp. | 1242 | 8.33 | 7.33 |
| *Bacillus* sp. | 1243 | 4.67 | 7.33 |
| *Agrococcus* sp. | 1244 | 11.67 | 9.67 |
| *Rhizobium* sp. | 1245 | 7.33 | 2.33 |
| *Variovorax* sp. | 1246 | 9.67 | 13.00 |
| *Rhizobium* sp. | 1249 | 6.00 | 10.00 |
| *Curtobacterium* sp. | 1250 | 3.67 | 6.00 |

| **Treatment** | **AIT ID** | **Repetition 1** | **Repetition 2** |
| --- | --- | --- | --- |
| Gibberellic acid |  | 5.33 | 1.67 |
| Maleic hydrazine |  | 0.33 | 3.00 |
| H₂O |  | 3.67 | 2.00 |
| 10% TSB |  | 3.67 | 2.67 |
| *Paenibacillus* sp. | 1187 | 3.33 | 2.33 |
| *Staphylococcus* sp. | 1213 | 4.33 | 2.67 |
| *Arthrobacter* sp. | 1214 | 3.00 | 2.00 |
| *Microbacterium* sp. | 1217 | 0.67 | 2.00 |
| *Arthrobacter* sp. | 1218 | 3.67 | 2.00 |
| *Staphylococcus* sp. | 1219 | 5.67 | 2.00 |
| *Variovorax* sp. | 1220 | 3.67 | 2.33 |
| *Arthrobacter* sp. | 1221 | 0.33 | 2.00 |
| *Arthrobacter* sp. | 1251 | 2.00 | 1.00 |
| *Microbacterium* sp. | 1252 | 1.67 | 2.00 |
| *Variovorax* sp. | 1254 | 1.33 | 0.67 |
| *Pedobacter* sp. | 1256 | 4.33 | 2.00 |
| *Arthrobacter* sp. | 1258 | 3.67 | 2.00 |
| *Rhodococcus* sp. | 1260 | 1.67 | 2.00 |
| *Arthrobacter* sp. | 1261 | 0.33 | 1.67 |
| *Microbacterium* sp. | 1262 | 2.67 | 1.67 |
| *Bacillus* sp. | 1263 | 0.00 | 1.00 |

| **Treatment** | **AIT ID** | **Repetition 1** | **Repetition 2** |
| --- | --- | --- | --- |
| Gibberellic acid |  | 12.33 | 7.67 |
| Maleic hydrazine |  | 0.00 | 0.33 |
| H₂O |  | 8.00 | 7.33 |
| 10% TSB |  | 5.00 | 6.33 |
| *Microbacterium* sp. | 1268 | 1.00 | 2.00 |
| *Arthrobacter* sp. | 1269 | 10.00 | 5.33 |
| *Streptomyces* sp. | 1278 | 4.33 | 8.00 |
| *Chryseobacterium* sp. | 1279 | 4.67 | 6.33 |
| *Pantoea* sp. | 1281 | 6.67 | 4.00 |
| *Bacillus* sp. | 1282 | 3.00 | 2.33 |
| *Chryseobacterium* sp. | 1283 | 4.00 | 2.33 |
| *Pantoea* sp. | 1284 | 3.67 | 2.67 |
| *Rhizobium* sp. | 1285 | 8.67 | 1.67 |
| *Streptomyces* sp. | 1286 | 11.33 | 4.67 |
| *Streptomyces* sp. | 1287 | 6.00 | 2.33 |
| *Bacillus* sp. | 1288 | 7.67 | 1.67 |
| *Bacillus* sp. | 1291 | 5.33 | 0.00 |
| *Achromobacter* sp. | 1293 | 6.33 | 5.67 |
| *Bacillus* sp. | 1294 | 5.33 | 2.33 |
| *Bacillus* sp. | 1296 | 6.33 | 8.33 |
| *Arthrobacter* sp. | 1299 | 8.33 | 4.67 |
| *Bacillus* sp. | 1301 | 4.00 | 4.00 |
| *Stenotrophomonas* sp. | 1302 | 5.67 | 4.00 |
| *Arthrobacter* sp. | 1303 | 8.67 | 7.33 |
| *Lysinibacillus* sp. | 1305 | 8.00 | 6.67 |
| *Paenibacillus* sp. | 1306 | 1.33 | 1.33 |
| *Bacillus* sp. | 1307 | 8.00 | 0.00 |
| *Paenibacillus* sp. | 1308 | 7.00 | 5.67 |

| **Treatment** | **AIT ID** | **Repetition 1** | **Repetition 2** |
| --- | --- | --- | --- |
| Gibberellic acid |  | 6.67 | 5.33 |
| Maleic hydrazine |  | 0.33 | 0.00 |
| H₂O |  | 2.33 | 3.33 |
| 10% TSB |  | 5.00 | 3.33 |
| *Arthrobacter* sp. | 1215 | 2.67 | 4.67 |
| *Brevundimonas* sp. | 1257 | 2.00 | 7.00 |
| *Bacillus* sp. | 1264 | 4.00 | 2.00 |
| *Bacillus* sp. | 1276 | 0.00 | 4.00 |
| *Devosia* sp. | 1277 | 7.33 | 8.67 |
| *Staphylococcus* sp. | 1280 | 6.33 | 5.33 |
| *Bacillus* sp. | 1304 | 4.33 | 6.33 |
| *Stenotrophomonas* sp. | 1309 | 3.00 | 0.00 |
| *Bacillus* sp. | 1311 | 2.00 | 5.33 |
| *Bacillus* sp. | 1312 | 4.67 | 3.33 |
| *Bacillus* sp. | 1314 | 5.33 | 5.33 |
| *Staphylococcus* sp. | 1315 | 0.00 | 0.00 |
| *Planococcaceae incertae sedis* | 1316 | 7.00 | 2.67 |
| *Bacillus* sp. | 1317 | 1.67 | 10.33 |
| *Roseomonas* sp. | 1318 | 0.33 | 1.33 |
| *Roseomonas* sp. | 1319 | 5.33 | 2.00 |
| *Enhydrobacter sp.* | 1320 | 2.67 | 3.00 |
| *Bacillus* sp. | 1322 | 2.67 | 2.67 |

| Treatment | **AIT ID** | Repetition 1 | Repetition 2 |
| --- | --- | --- | --- |
| Gibberellic acid |  | 8.00 | 7.33 |
| Maleic hydrazine |  | 0.00 | 0.33 |
| H₂O |  | 7.33 | 5.33 |
| 10% TSB |  | 5.33 | 3.67 |
| *Lysinibacillus* sp. | 1323 | 0.67 | 0.00 |
| *Kocuria* sp. | 1222 | 1.33 | 3.33 |
| *Novosphingobium* sp. | 1354 | 3.00 | 2.67 |
| *Rhodococcus* sp. | 1355 | 0.00 | 4.00 |
| *Arthrobacter* sp. | 1357 | 2.67 | 5.67 |
| *Microbacterium* sp. | 1358 | 2.33 | 0.33 |
| *Bacillus* sp. | 1359 | 3.67 | 4.00 |
| *Bacillus* sp. | 1360 | 0.67 | 1.33 |
| *Bacillus* sp. | 1361 | 1.00 | 2.33 |
| *Arthrobacter* sp. | 1362 | 2.00 | 2.33 |
| *Arthrobacter* sp. | 1363 | 0.00 | 0.67 |
| *Arthrobacter* sp. | 1364 | 0.00 | 1.33 |
| *Arthrobacter* sp. | 1366 | 2.00 | 0.00 |
| *Arthrobacter* sp. | 1367 | 1.00 | 2.00 |
| *Microbacterium* sp. | 1368 | 1.00 | 1.00 |
| *Arthrobacter* sp. | 1369 | 1.33 | 0.00 |
| *Bacillus* sp. | 1371 | 4.00 | 2.33 |
| *Mycobacterium* sp. | 1372 | 4.00 | 3.00 |
| *Bacillus* sp. | 1373 | 2.33 | 4.00 |
| *Bacillus* sp. | 1374 | 3.33 | 1.33 |
| *Bacillus* sp. | 1375 | 3.00 | 0.33 |
| *Bacillus* sp. | 1376 | 0.00 | 1.33 |
| *Bacillus* sp. | 1377 | 2.00 | 0.00 |
| *Bacillus* sp. | 1378 | 1.67 | 4.67 |
| *Rhizobium* sp. | 1379 | 1.00 | 4.67 |
| *Microbacterium* sp. | 1380 | 0.00 | 2.33 |

| **Treatment** | **AIT ID** | **Repetition 1** | **Repetition 2** |
| --- | --- | --- | --- |
| Gibberellic acid |  | 4.00 | 4.33 |
| Maleic hydrazine |  | 0.00 | 0.00 |
| H₂O |  | 1.67 | 5.33 |
| 10% TSB |  | 6.00 | 2.67 |
| *Arthrobacter* sp. | 1321 | 1.33 | 0.33 |
| *Lysinibacillus* sp. | 1324 | 1.33 | 3.00 |
| *Arthrobacter* sp. | 1326 | 0.00 | 0.33 |
| *Arthrobacter* sp. | 1328 | 1.33 | 3.67 |
| *Massilia* sp. | 1329 | 3.00 | 4.67 |
| *Bacillus* sp. | 1330 | 3.00 | 1.67 |
| *Paenibacillus* sp. | 1331 | 3.00 | 1.67 |
| *Staphylococcus* sp. | 1332 | 2.00 | 0.00 |
| *Bacillus* sp. | 1334 | 4.67 | 0.00 |
| *Arthrobacter* sp. | 1335 | 0.00 | 0.00 |
| *Arthrobacter* sp. | 1338 | 0.00 | 2.33 |
| *Phycicoccus* sp. | 1339 | 1.67 | 0.33 |
| Staphylococcus sp. | 1340 | 1.67 | 0.00 |
| *Paenibacillus* sp. | 1341 | 2.00 | 0.33 |
| *Paenibacillus* sp. | 1342 | 0.33 | 0.33 |
| *Staphylococcus* sp. | 1343 | 1.00 | 0.33 |
| *Sphingomonas* sp. | 1350 | 3.67 | 7.67 |
| *Paenibacillus* sp. | 1272 | 0.33 | 3.33 |
| *Arthrobacter* sp. | 1381 | 1.67 | 3.00 |
| *Arthrobacter* sp. | 1382 | 3.33 | 2.67 |
| *Bacillus* sp. | 1383 | 1.67 | 3.00 |
| *Bacillus* sp. | 1385 | 0.00 | 0.33 |
| *Microbacterium* sp. | 1386 | 0.33 | 2.00 |
| *Bacillus* sp. | 1387 | 1.00 | 1.00 |
| *Arthrobacter* sp. | 1388 | 1.00 | 0.33 |

**Supplementary Table 11:** **Results of the statistical analysis of the *in-vitro* potato tuber sprouting assay.** P values show significant differences between treatments with bacterial isolates and the assay control 10% sterile tryptic soy broth calculated for each sprouting assay repetition.

| **AIT ID** | **Taxa** | **P value Repetition 1** | **P value Repetition 2** |
| --- | --- | --- | --- |
| 1125 | *Microbacterium* sp. | 0.04 | 0.01 |
| 1126 | *Bacillus* sp. | 0.04 | 0.01 |
| 1136 | *Arthrobacter* sp. | 0.04 | 0.01 |
| 1138 | *Bacillus* sp. | 0.04 | 0.01 |
| 1143 | *Arthrobacter* sp. | 0.04 | 0.01 |
| 1144 | *Stenotrophomonas* sp. | 0.04 | 0.01 |
| 1146 | *Bacillus* sp. | 0.04 | 0.01 |
| 1149 | *Bacillus* sp. | 0.04 | 0.01 |
| 1151 | *Microbacterium* sp. | 0.04 | 0.01 |
| 1184 | *Bacillus* sp. | 0.00 | 0.00 |

**Supplementary Table S12:** **Primers used for the amplification of region V5-V7 of the 16S rRNA gene of the potato tuber bacterial communities.** Sample-specific indices are shown in red.

| **Primer name** | | **Oligo Sequence (5´- 3´)** | |
| --- | --- | --- | --- |
| 16S rRNA PCR round 1 | | | |
| 799f | | AACMGGATTAGATACCCKG | |
| 1175r | | ACGTCRTCCCCDCCTTCCT | |
| 16S rRNA PCR round 2 | | | |
| **Primer name** | **Oligo Sequence (5´- 3´)** | **Primer name** | **Oligo Sequence (5´- 3´)** |
| 799F_1 | ACAACCAGTTAACMGGATTAGATACCCKG | 1175R_1 | ACAACCAGTTACGTCRTCCCCDCCTTCCT |
| 799F_2 | NAACAGACCTTAACMGGATTAGATACCCKG | 1175R_2 | NAACAGACCTTACGTCRTCCCCDCCTTCCT |
| 799F_3 | NNACAAGGTCTTAACMGGATTAGATACCCKG | 1175R_3 | NNACAAGGTCTTACGTCRTCCCCDCCTTCCT |
| 799F_4 | NNNAAGTCTTCGTAACMGGATTAGATACCCKG | 1175R_4 | NNNAAGTCTTCGTACGTCRTCCCCDCCTTCCT |
| 799F_5 | ACATGAGGTTAACMGGATTAGATACCCKG | 1175R_5 | ACATGAGGTTACGTCRTCCCCDCCTTCCT |
| 799F_6 | NAAGCTCACTTAACMGGATTAGATACCCKG | 1175R_6 | NAAGCTCACTTACGTCRTCCCCDCCTTCCT |
| 799F_7 | NNACGATACGTTAACMGGATTAGATACCCKG | 1175R_7 | NNACGATACGTTACGTCRTCCCCDCCTTCCT |
| 799F_8 | NNNAATGCGCTATAACMGGATTAGATACCCKG | 1175R_8 | NNNAATGCGCTATACGTCRTCCCCDCCTTCCT |
| 799F_9 | ACCTCATCTTAACMGGATTAGATACCCKG | 1175R_9 | ACCTCATCTTACGTCRTCCCCDCCTTCCT |
| 799F_10 | NAACAGCTCATAACMGGATTAGATACCCKG | 1175R_10 | NAACAGCTCATACGTCRTCCCCDCCTTCCT |
| 799F_11 | NNACTGTTGACTAACMGGATTAGATACCCKG | 1175R_11 | NNACTGTTGACTACGTCRTCCCCDCCTTCCT |
| 799F_12 | NNNAGAGTTGCTTAACMGGATTAGATACCCKG | 1175R_12 | NNNAGAGTTGCTTACGTCRTCCCCDCCTTCCT |
| 799F_13 | ACCTTGACATAACMGGATTAGATACCCKG | 1175R_13 | ACCTTGACATACGTCRTCCCCDCCTTCCT |
| 799F_14 | NACAACTGTGTAACMGGATTAGATACCCKG | 1175R_14 | NACAACTGTGTACGTCRTCCCCDCCTTCCT |
| 799F_15 | NNACTTAGCACTAACMGGATTAGATACCCKG | 1175R_15 | NNACTTAGCACTACGTCRTCCCCDCCTTCCT |
| 799F_16 | NNNAATACGACCTAACMGGATTAGATACCCKG | 1175R_16 | NNNAATACGACCTACGTCRTCCCCDCCTTCCT |
| 799F_17 | ACGATCGTATAACMGGATTAGATACCCKG | 1175R_17 | ACGATCGTATACGTCRTCCCCDCCTTCCT |
| 799F_18 | NACCAATCAGTAACMGGATTAGATACCCKG | 1175R_18 | NACCAATCAGTACGTCRTCCCCDCCTTCCT |
| 799F_19 | NNAGGACTTGTTAACMGGATTAGATACCCKG | 1175R_19 | NNAGGACTTGTTACGTCRTCCCCDCCTTCCT |
| 799F_20 | NNNAGCTGAATCTAACMGGATTAGATACCCKG | 1175R_20 | NNNAGCTGAATCTACGTCRTCCCCDCCTTCCT |
| 799F_21 | ACTCACTGTTAACMGGATTAGATACCCKG | 1175R_21 | ACTCACTGTTACGTCRTCCCCDCCTTCCT |
| 799F_22 | NAGAGCAATGTAACMGGATTAGATACCCKG | 1175R_22 | NAGAGCAATGTACGTCRTCCCCDCCTTCCT |
| 799F_23 | NNAGTCATCCTTAACMGGATTAGATACCCKG | 1175R_23 | NNAGTCATCCTTACGTCRTCCCCDCCTTCCT |
| 799F_24 | NNNAGTAGCCTATAACMGGATTAGATACCCKG | 1175R_24 | NNNAGTAGCCTATACGTCRTCCCCDCCTTCCT |
| 799F_25 | AGATAGCGATAACMGGATTAGATACCCKG | 1175R_25 | AGATAGCGATACGTCRTCCCCDCCTTCCT |
| 799F_26 | NAACGGAACATAACMGGATTAGATACCCKG | 1175R_26 | NAACGGAACATACGTCRTCCCCDCCTTCCT |
| 799F_27 | NNATATAGCCGTAACMGGATTAGATACCCKG | 1175R_27 | NNATATAGCCGTACGTCRTCCCCDCCTTCCT |
| 799F_28 | NNNAGTGAACTCTAACMGGATTAGATACCCKG | 1175R_28 | NNNAGTGAACTCTACGTCRTCCCCDCCTTCCT |
| 799F_29 | ATACGGACTTAACMGGATTAGATACCCKG | 1175R_29 | ATACGGACTTACGTCRTCCCCDCCTTCCT |
| 799F_30 | NAGCTCCTTATAACMGGATTAGATACCCKG | 1175R_30 | NAGCTCCTTATACGTCRTCCCCDCCTTCCT |
| 799F_31 | NNATGCGCATATAACMGGATTAGATACCCKG | 1175R_31 | NNATGCGCATATACGTCRTCCCCDCCTTCCT |
| 799F_32 | NNNAGTGCTTCATAACMGGATTAGATACCCKG | 1175R_32 | NNNAGTGCTTCATACGTCRTCCCCDCCTTCCT |
| 799F_33 | ATCACCATGTAACMGGATTAGATACCCKG | 1175R_33 | ATCACCATGTACGTCRTCCCCDCCTTCCT |
| 799F_34 | NAGCTTCAGTTAACMGGATTAGATACCCKG | 1175R_34 | NAGCTTCAGTTACGTCRTCCCCDCCTTCCT |
| 799F_35 | NNATGTACTGGTAACMGGATTAGATACCCKG | 1175R_35 | NNATGTACTGGTACGTCRTCCCCDCCTTCCT |
| 799F_36 | NNNATCGGTAGTTAACMGGATTAGATACCCKG | 1175R_36 | NNNATCGGTAGTTACGTCRTCCCCDCCTTCCT |
| 799F_37 | ATCGAACCTTAACMGGATTAGATACCCKG | 1175R_37 | ATCGAACCTTACGTCRTCCCCDCCTTCCT |
| 799F_38 | NAGGTACAACTAACMGGATTAGATACCCKG | 1175R_38 | NAGGTACAACTACGTCRTCCCCDCCTTCCT |
| 799F_39 | NNATTGGAGTGTAACMGGATTAGATACCCKG | 1175R_39 | NNATTGGAGTGTACGTCRTCCCCDCCTTCCT |
| 799F_40 | NNNAGACATTCCTAACMGGATTAGATACCCKG | 1175R_40 | NNNAGACATTCCTACGTCRTCCCCDCCTTCCT |
| 799F_41 | ATGCAACACTAACMGGATTAGATACCCKG | 1175R_41 | ATGCAACACTACGTCRTCCCCDCCTTCCT |
| 799F_42 | NAGGTGTGTTTAACMGGATTAGATACCCKG | 1175R_42 | NAGGTGTGTTTACGTCRTCCCCDCCTTCCT |
| 799F_43 | ATGGTAACGTAACMGGATTAGATACCCKG | 1175R_43 | ATGGTAACGTACGTCRTCCCCDCCTTCCT |
| 799F_44 | NAGTCGATACTAACMGGATTAGATACCCKG | 1175R_44 | NAGTCGATACTACGTCRTCCCCDCCTTCCT |
| 799F_45 | ATTCACCTGTAACMGGATTAGATACCCKG | 1175R_45 | ATTCACCTGTACGTCRTCCCCDCCTTCCT |
| 799F_46 | NAGTTGAGCATAACMGGATTAGATACCCKG | 1175R_46 | NAGTTGAGCATACGTCRTCCCCDCCTTCCT |
| 799F_47 | ATTGACACCTAACMGGATTAGATACCCKG | 1175R_47 | ATTGACACCTACGTCRTCCCCDCCTTCCT |
| 799F_48 | NATACGTTGCTAACMGGATTAGATACCCKG | 1175R_48 | NATACGTTGCTACGTCRTCCCCDCCTTCCT |


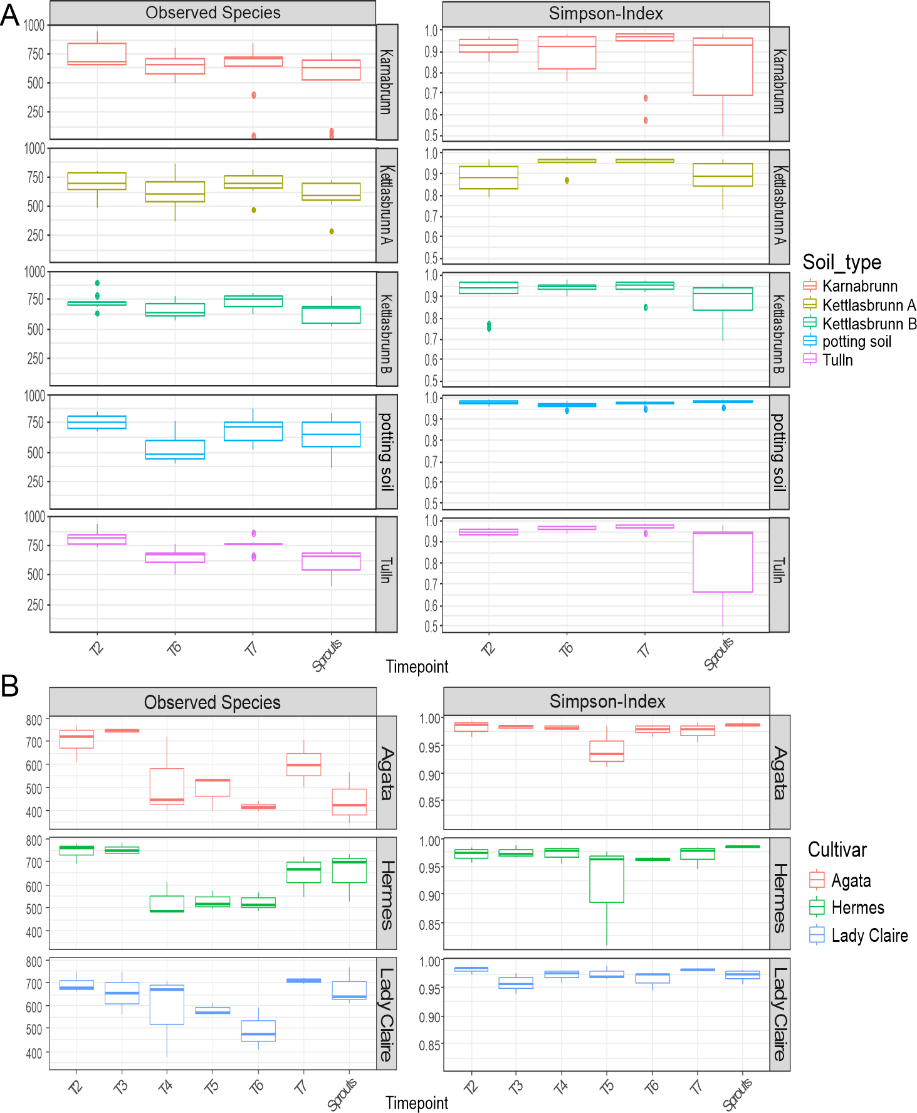


**Supplementary Figure S1: Alpha diversity as measured by bacterial richness and Simpson index.** Values are visualized by boxplots to provide a comprehensive assessment of bacterial diversity for **A.**, tubers of the variety (Agata, Lady Claire and Hermes) sampled at T2, T6 and T7 and **B.** tubers of the variety (Agata, Lady Claire and Hermes) sampled at T2-7 and sprouts at T7. An overview of potato cultivars, soil types and sampling time points is shown in Figure 1.


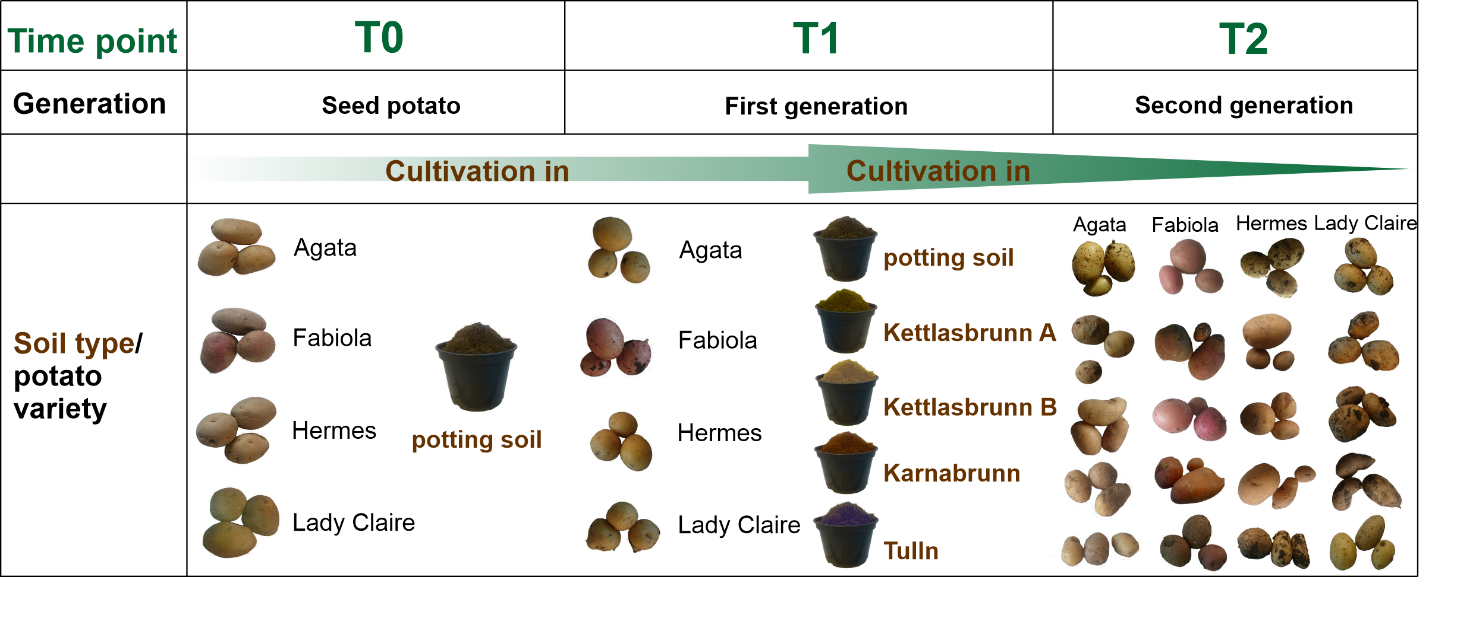


**Supplementary Figure S2:** **Overview of potato cultivars and tuber generation investigated in a previous study**[**^19^**](#_ENREF_19)**.** Initially, seed potatoes of four potato cultivars (Agata, Fabiola, Lady Claire and Hermes) were used for 16S rRNA gene amplicon sequencing (T0) and were grown in parallel in commercial potting soil. At maturity, tubers were harvested and used for 16S rRNA gene amplicon sequencing (T1). Tubers were planted in pots with five different soil types (commercial potting soil and four different farmland soils). Again, tubers were harvested at maturity and used for bacterial community sequencing (T2).


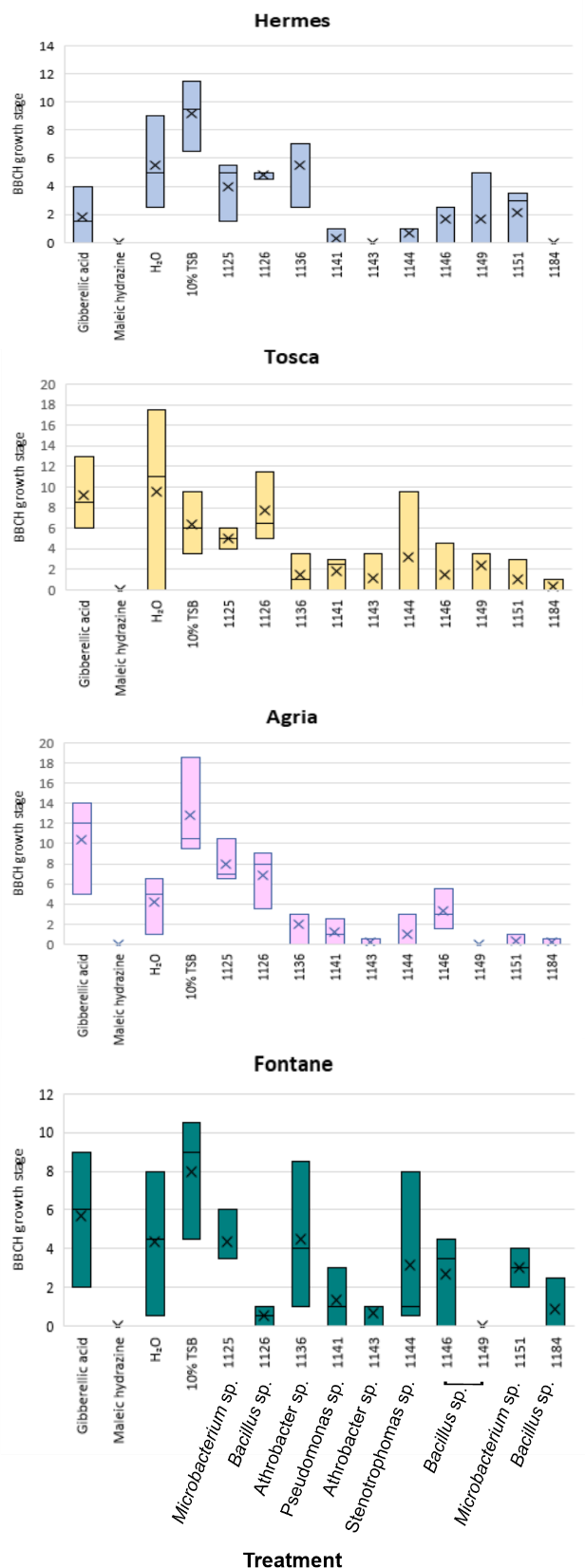


**Supplementary Figure S3: *In-vitro* potato tuber sprouting assay results of selected bacterial isolates.** Tuber buds of four different varieties (Hermes, Tosca, Fontane and Agria) treated with ten selected isolate cultures. When tuber buds treated with maleic hydrazine reached stage 01, the assay was closed and the sum of all growth stages of each replicate was calculated. Afterwards the average of two repetitions was visualized in a boxplot diagram. The boxplot diagram shows that treatments with cultures of *Bacillus* sp. (AIT 1184) and *Arthrobacter* sp. (AIT 1143) led to a sprouting inhibition in comparison to the assay control 10% sterile tryptic soy broth.

**Supplementary Methods**

***16S rRNA gene amplicon sequence processing***

After sequencing, quality of MiSeq raw data was checked in FastQC[^1^](file:///C:\Users\Franziska\Desktop\Manuscript%20Buchholz%20et%20al%20revised%20.docx#_ENREF_47) and reads were screened for PhiX contamination by using Bowti 2.2.6[^2^](file:///C:\Users\Franziska\Desktop\Manuscript%20Buchholz%20et%20al%20revised%20.docx#_ENREF_48). Samples were demultiplexed and primers were stripped out using Cutadapt 1.8.3[^3^](file:///C:\Users\Franziska\Desktop\Manuscript%20Buchholz%20et%20al%20revised%20.docx#_ENREF_49). Paired-end reads were then merged employing PEAR 0.9.10 (p< 0.001)[^4^](file:///C:\Users\Franziska\Desktop\Manuscript%20Buchholz%20et%20al%20revised%20.docx#_ENREF_50) and quality filtering was performed in USEARCH v8.0.1517 (maximum expected error=0.5)^5,^[^6^](file:///C:\Users\Franziska\Desktop\Manuscript%20Buchholz%20et%20al%20revised%20.docx#_ENREF_52). METAXA2.2 was used to discard data other than archaeal and bacterial sequences and to target the extraction of the 16S rRNA V5-V7 gene region from filtered reads[^7^](file:///C:\Users\Franziska\Desktop\Manuscript%20Buchholz%20et%20al%20revised%20.docx#_ENREF_53). In QIIME 1.9.1, all targeted reads were attached to their sample name of origin and combined[^8^](file:///C:\Users\Franziska\Desktop\Manuscript%20Buchholz%20et%20al%20revised%20.docx#_ENREF_54). Then VSEARCH 1.1.1 was used to de-replicate, to sort and to cluster sequences at 97% of similarity[^9^](file:///C:\Users\Franziska\Desktop\Manuscript%20Buchholz%20et%20al%20revised%20.docx#_ENREF_55). A *de nov*o and a reference-based approach was used to check for chimeras as a routine of the above-mentioned tool. As database for the reference-based chimera detection, the SILVA database v.128 (97% OTU repset) was used. A BIOM table was built after an optimal alignment was applied in VSEARCH. The taxonomy assignment was performed after using the naïve Bayesian RDP classifier v2.10[^10^](file:///C:\Users\Franziska\Desktop\Manuscript%20Buchholz%20et%20al%20revised%20.docx#_ENREF_56) in QIIME with a minimum confidence of 0.8 against the SILVA database release 128^11,^[^12^](file:///C:\Users\Franziska\Desktop\Manuscript%20Buchholz%20et%20al%20revised%20.docx#_ENREF_58).

***Statistics analysis***

After building the OTU table in QIIME, sequences were processed and analysed with the R v3.6.2 software environment[^13^](file:///C:\Users\Franziska\Desktop\Manuscript%20Buchholz%20et%20al%20revised%20.docx#_ENREF_45). Therefore, samples with less than > 0.01% relative abundance were filtered by applying the filter.OTU function of the R package RAM[^14^](file:///C:\Users\Franziska\Desktop\Manuscript%20Buchholz%20et%20al%20revised%20.docx#_ENREF_59). To determine if OTUs were consistently found among three replicates of a sample collection, ‘reproducibly occurring OTU’ were defined with the RAM::rep.OTU function. Only OTUs, which were detected in at least 66.66% (2 out of 3 replicates), were considered for a further analysis.

For alpha values calculation of observed species and Simpson’s index, the rtk package was used after a multiple rarefaction and averaging the results of 9999 iterations[^15^](file:///C:\Users\Franziska\Desktop\Manuscript%20Buchholz%20et%20al%20revised%20.docx#_ENREF_60). Afterwards, observed rOTUs were calculated and Simpson’s diversity index was applied to estimate the diversity within each individual sample. Richness and diversity values were then compared between time points, between varieties and between soil types by means of permutation ANOVA (i. e., perm.anova) and permutation pairwise comparison (i. e., pairwise.perm.t.test) function in the RVAideMemoire R package[^16^](file:///C:\Users\Franziska\Desktop\Manuscript%20Buchholz%20et%20al%20revised%20.docx#_ENREF_61). To correct for multiple testing, all P-values were adjusted by False Discovery Rate (FDR). Richness and diversity values were visualized with the help of the RAM::group.diversity function in a boxplots. For beta diversity calculations, a normalization method based on cumulative sum scaling (CSS) was applied[^17^](file:///C:\Users\Franziska\Desktop\Manuscript%20Buchholz%20et%20al%20revised%20.docx#_ENREF_62) to remove biases in the count data. This was done with the metagenomeSeq Bioconductor package[^18^](file:///C:\Users\Franziska\Desktop\Manuscript%20Buchholz%20et%20al%20revised%20.docx#_ENREF_63). As an ordination method the Bray-Curtis dissimilarity distance was calculated to determine the differences between bacterial communities. Permutation test for assessing the significance of the permutational multivariate analysis of variances (PERMANOVA) was done. Therefore, the null hypothesis of no differences between a priori defined groups like time point, soil type and cultivar was studied recurring the function adonis of the vegan R package[^19^](file:///C:\Users\Franziska\Desktop\Manuscript%20Buchholz%20et%20al%20revised%20.docx#_ENREF_64) to the Bray-Curtis dissimilarity distance.

The applicability of the PERMANOVA was checked with the analysis of the multivariate homogeneity of group dispersion of each grouping factor by using the vegan::betadisper function. A generalized linear model for multivariate abundance data was applied with the manyglm function of the mvabund R package[^20^](file:///C:\Users\Franziska\Desktop\Manuscript%20Buchholz%20et%20al%20revised%20.docx#_ENREF_65) because multivariate homogeneity was not present in all grouping factors to confirm results from PERMANOVA. Correlation and individuation of rOTUs responsible for shaping the diversity structure was done by calculating the permutational pairwise comparison with the RVAideMemoire::pairwise.perm.manova between the grouping factors.

The multivariate analysis of community structure and diversity was achieved by doing a constrained multidimensional scaling using Constrained Analysis of Principle Coordinates which was computed and plotted in the phyloseq R package[^21^](file:///C:\Users\Franziska\Desktop\Manuscript%20Buchholz%20et%20al%20revised%20.docx#_ENREF_66). The significance of the grouping factor time point, soil type and cultivar used as constrained a CAP was performed with the permutation test implemented in the vegan::capscale function. Calculation of differential abundant rOTUs with each time point and/or separated by storage time was performed with the random forest function of the same R package[^22^](file:///C:\Users\Franziska\Desktop\Manuscript%20Buchholz%20et%20al%20revised%20.docx#_ENREF_67). Differentially abundant rOTUs were then selected with the function varSelRF of the same R package[^23^](file:///C:\Users\Franziska\Desktop\Manuscript%20Buchholz%20et%20al%20revised%20.docx#_ENREF_68) and visualized in a bar plot with the function RAM::group.abundant.Taxa.

**References**

1 Andrew, S. FastQC: a quality control tool for high throughput sequence data. http://www.bioinformatics.babraham.ac.uk/projects/fastqc (2010).

2 Langmead, B. & Salzberg, S. L. Fast gapped-read alignment with Bowtie 2. *Nat. Methods* **9**, 357-359, doi:10.1038/nmeth.1923 (2012).

3 Martin, M. Cutadapt Removes Adapter Sequences from High-Throughput Sequencing Reads. *EMBnet Journal* **17**, 10-12 (2018).

4 Zhang, J., Kobert, K., Flouri, T. & Stamatakis, A. PEAR: a fast and accurate Illumina Paired-End reAd mergeR. *Bioinformatics* **30**, 614-620, doi:10.1093/bioinformatics/btt593 (2014).

5 Edgar, R. C. UPARSE: highly accurate OTU sequences from microbial amplicon reads. *Nat. Methods* **10**, 996, doi:10.1038/nmeth.2604 (2013).

6 Edgar, R. C. & Flyvbjerg, H. Error filtering, pair assembly and error correction for next-generation sequencing reads. *Bioinformatics* **31**, 3476-3482, doi:10.1093/bioinformatics/btv401 (2015).

7 Bengtsson-Palme, J. *et al.* metaxa2: improved identification and taxonomic classification of small and large subunit rRNA in metagenomic data. *Molecular Ecology Resources* **15**, 1403-1414, doi:10.1111/1755-0998.12399 (2015).

8 Caporaso, J. G. *et al.* QIIME allows analysis of high-throughput community sequencing data. *Nat. Methods* **7**, 335-336, doi:10.1038/nmeth.f.303 (2010).

9 Rognes, T. VSEARCH: versatile open-source tool for metagenomics. https://github.com/torognes/vsearch (2015).

10 Wang, Q., Garrity, G. M., Tiedje, J. M. & Cole, J. R. Naïve Bayesian Classifier for Rapid Assignment of rRNA Sequences into the New Bacterial Taxonomy. *Appl. Environ. Microbiol.* **73**, 5261-5267, doi:10.1128/AEM.00062-07 (2007).

11 Quast, C. *et al.* The SILVA ribosomal RNA gene database project: improved data processing and web-based tools. *Nucleic Acids Res.* **41**, D590-D596, doi:10.1093/nar/gks1219 (2013).

12 Schirmer, M. *et al.* Insight into biases and sequencing errors for amplicon sequencing with the Illumina MiSeq platform. *Nucleic Acids Res.* **43**, e37-e37, doi:10.1093/nar/gku1341 (2015).

13 R Core Team. R: A language and environment for statistical computing. http://www.R-project.org/ (2013).

14 Chen, W., Simpson, C. & Levesque, A. RAM: R for Amplicon-Sequencing-Based Microbial-Ecology. R package version 1.2.1.7. https://cran.r-project.org/web/packages/RAM/RAM.pdf (2018).

15 Saary, P., Forslund, K., Bork, P. & Hildebrand, F. RTK: efficient rarefaction analysis of large datasets. R package version 0.2.5.7. doi:doi: 10.1093/bioinformatics/btx206 (2017).

16 Hervé, M. RVAideMemoire: Testing and Plotting Procedures for Biostatistics. R package version 0.9-69-3. https://cran.r-project.org/web/packages/RVAideMemoire/RVAideMemoire.pdf. (2019).

17 Paulson, J. N., Stine, O. C., Bravo, H. C. & Pop, M. Differential abundance analysis for microbial marker-gene surveys. *Nat. Methods* **10**, 1200-1202, doi:10.1038/nmeth.2658 (2013).

18 Paulson, J. *et al.* metagenomSeq: Statistical analysis for sparse high-throughput sequencing. R package Version 1.24.1. https://github.com/nosson/metagenomeSeq/ (2019).

19 Oksanen, J. *et al.* vegan: Community Ecology Package. R Package. Version 2. 5-2. https://CRAN.R-project.org/package=vegan (2018).

20 Wang Y., Naumann U., Wright S. & Warton D. mvabund: an R package for model-based analysis of multivariate data.R package version 3.6.11. *Methods in Ecology & Evolution* **3**, 471-474 (2012).

21 McMurdie, P. J. & Holmes, S. phyloseq: An R Package for Reproducible Interactive Analysis and Graphics of Microbiome Census Data. *PLoS ONE* **8**, e61217, doi:10.1371/journal.pone.0061217 (2013).

22 Liaw, A. & Wiener, M. *Classification and Regression by RandomForest*. Vol. 23 (2001).

23 Diaz-Uriarte, R. GeneSrF and varSelRF: a web-based tool and R package for gene selection and classification using random forest. *BMC Bioinformatics* **8**, 328, doi:10.1186/1471-2105-8-328 (2007).
